# High-Performance Flexible Microneedle Array as a Low-Impedance Surface Biopotential Dry Electrode for Wearable Electrophysiological Recording and Polysomnography

**DOI:** 10.1007/s40820-022-00870-0

**Published:** 2022-06-14

**Authors:** Junshi Li, Yundong Ma, Dong Huang, Zhongyan Wang, Zhitong Zhang, Yingjie Ren, Mengyue Hong, Yufeng Chen, Tingyu Li, Xiaoyi Shi, Lu Cao, Jiayan Zhang, Bingli Jiao, Junhua Liu, Hongqiang Sun, Zhihong Li

**Affiliations:** 1 National Key Laboratory of Science and Technology on Micro/Nano Fabrication, School of Integrated Circuits, Peking University, Beijing, 100871 People’s Republic of China; 2 Peking University Sixth Hospital, Peking University Institute of Mental Health, NHC Key Laboratory of Mental Health (Peking University), National Clinical Research Center for Mental Disorders (Peking University Sixth Hospital), Beijing, 100191 People’s Republic of China; 3 School of Electronics, Peking University, Beijing, 100871 People’s Republic of China; 4 Hypnometry Microsystem, Beijing, 100871 People’s Republic of China; 5 College of Engineering, Peking University, Beijing, 100871 People’s Republic of China

**Keywords:** Flexible microneedle array, Dry electrode, Low-impedance electrode–skin contact, Wearable wireless electrophysiological recording, Polysomnography

## Abstract

**Highlights:**

- Polyimide-based flexible microneedle array (PI-MNA) electrodes realize high electrical/mechanical performance and are compatible with wearable wireless recording systems.
- The normalized electrode–skin interface impedance (EII) of the PI-MNA electrodes reaches 0.98 kΩ cm^2^ at 1 kHz and 1.50 kΩ cm^2^ at 10 Hz, approximately 1/250 of clinical standard electrodes.
- This is the first report on the clinical study of microneedle electrodes. The PI-MNA electrodes are applied to clinical long-term continuous monitoring for polysomnography.

**Abstract:**

Microneedle array (MNA) electrodes are an effective solution to achieve high-quality surface biopotential recording without the coordination of conductive gel and are thus very suitable for long-term wearable applications. Existing schemes are limited by flexibility, biosafety, and manufacturing costs, which create large barriers for wider applications. Here, we present a novel flexible MNA electrode that can simultaneously achieve flexibility of the substrate to fit a curved body surface, robustness of microneedles to penetrate the skin without fracture, and a simplified process to allow mass production. The compatibility with wearable wireless systems and the short preparation time of the electrodes significantly improves the comfort and convenience of electrophysiological recording. The normalized electrode–skin contact impedance reaches 0.98 kΩ cm^2^ at 1 kHz and 1.50 kΩ cm^2^ at 10 Hz, a record low value compared to previous reports and approximately 1/250 of the standard electrodes. The morphology, biosafety, and electrical/mechanical properties are fully characterized, and wearable recordings with a high signal-to-noise ratio and low motion artifacts are realized. The first reported clinical study of microneedle electrodes for surface electrophysiological monitoring was conducted in tens of healthy and sleep-disordered subjects with 44 nights of recording (over 8 h per night), providing substantial evidence that the electrodes can be leveraged to substitute for clinical standard electrodes.
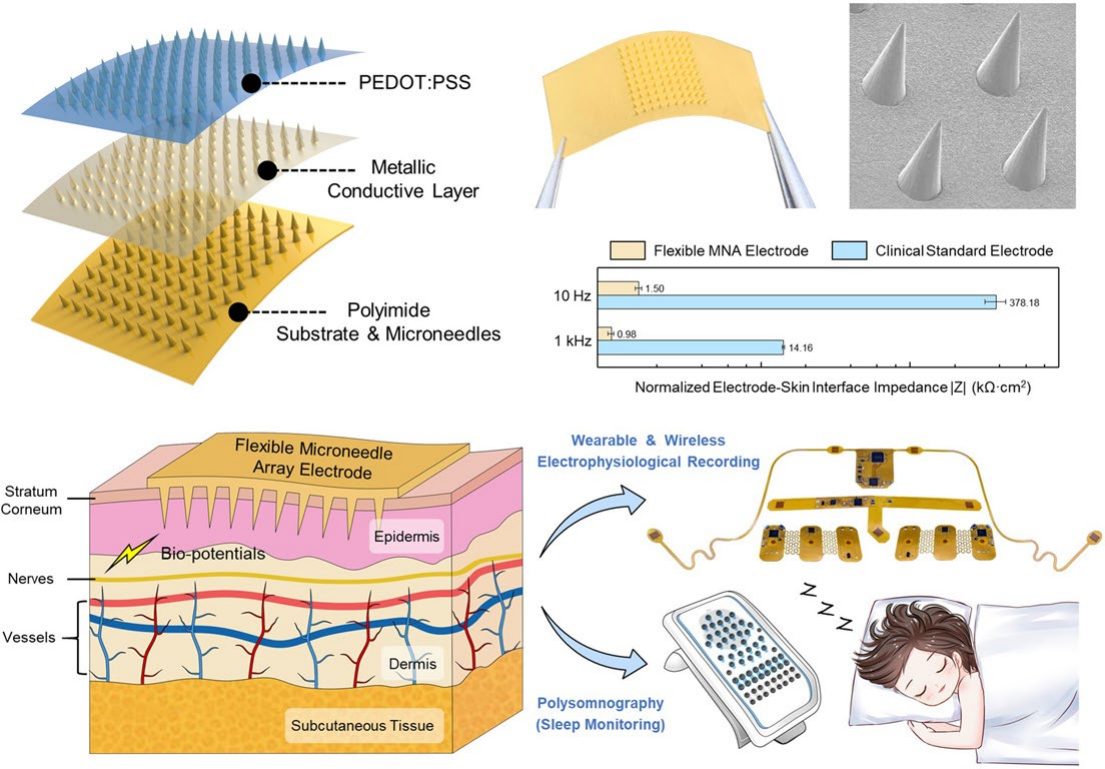

**Supplementary Information:**

The online version contains supplementary material available at 10.1007/s40820-022-00870-0.

## Introduction

Surface biopotentials are key indicators for physical function, mental condition, and pathological diagnosis [1–3]. Based on advances in bioelectric acquisition technology, electrocardiography (ECG), electromyography (EMG), electroencephalography (EEG), and electrooculography (EOG) have been generally used in clinical practice. Meanwhile, with the development of biopotential electrodes and artificial intelligence algorithm, surface biopotentials have been increasingly employed for human–machine interfaces as a noninvasive solution [4]. As electrodes are worn on skin for biopotential acquisition, their essence is the transducers that convert ionic current from the human body into electronic current that can be input into an external electronic system [5]. The electrode–skin interface impedance (EII) is a crucial parameter [1]. A lower EII means higher signal quality, a higher signal-to-noise ratio (SNR), and less baseline drift.

Conventional biopotential electrodes, which are generally called wet electrodes, must be used with conductive gel to moisten the skin and create low-resistance conductive channels. Existing instruments for electrophysiological monitoring are connected with wet electrodes in a wired way. For clinical applications with multiple channels, such as multilead ECG/EEG, dynamic electrophysiological monitoring (Holter), and polysomnography (PSG), patients have to wear a large number of complicated wires. The bulky structure, large footprint, liquid conductive gel, and mass wiring severely restrict the convenience and free movement of patients. Moreover, the heavy instrument and mass wires also put the electrodes at the risk of becoming loose or falling off during movements. For example, conventional bedside PSG instrument (Fig. S1a) can seriously affect the sleep quality of patients who already have sleep disorders. Continuous monitoring for several nights is often required to reflect the real sleep status, and introduces excess time and economic costs to both patients and hospitals. Portable PSG instruments (Fig. S1b), which have gained more acceptance in recent years, allow patients to walk freely with the instruments and wear them home for sleep monitoring. Even so, wires attached to the patient do not decrease, and the instrument should press against the body during sleep; the sleep quality will still be inevitably disrupted. In addition, for weak signals such as EEG, proper skin preparation is indispensable to thin the high-resistance stratum corneum (SC). To achieve good contact throughout the night, skin around the recording sites needs to be treated with scrub cream before applying the electrodes and conductive gel (Fig. S2). A skilled sleep physician usually needs to take more than 30 min to attach all the electrodes to a patient. When sleep monitoring ends, additional dozens of minutes are needed to disconnect the electrodes and remove the conductive gel. Thus, the procedure for wearing and removing electrodes is very tedious.

Dry electrodes without conductive gel are better suited for long-term electrophysiological monitoring. However, due to the highly resistive SC, dry electrodes generally do not exhibit good EII compared to wet electrodes [6, 7]. Various new materials and structures have been proposed to improve the electrode–skin contact, but the trade-off is the large electrode volume or footprint [3–5, 8–10].

Microneedle arrays (MNAs) have demonstrated excellent performance in bioelectrode, neural interface, drug delivery, electroosmotic, and biochemical sensing applications [11–24]. Researchers have proposed diverse MNA devices with different heights, angles, sharpness, and densities [13]. Special microneedle structures inspired by nature, such as of eagle claws, snake fangs, honeybees stingers, and mosquito proboscises, have been designed to achieve more efficient skin fitness, tissue adhesion, and special functionalities, such as healing promotion and painless blood collection [24]. MNAs as dry electrodes for surface biopotential recording can realize low-impedance contact, as the spiky tips are able to penetrate SC [6, 13, 25–27]. Using microneedle electrodes can skip the skin preparation procedure; thus, the preparation time before wearing electrodes is reduced and not sensitive to the condition of the skin surface, which is significantly convenient for both patients and operators. Currently, microneedle electrodes proposed by researchers are pursuing a smaller footprint, better mechanical match with skin (*i.e.*, flexibility), and higher biocompatibility [28–32]. Smaller electrodes are easier to attach to skin with medical tape for greater comfort and safety, and allow higher spatial resolution to form a high-density recording array. Meanwhile, the SNR is also related to the contact area because the skin surface is distributed with various electrophysiological signals from different sources. A smaller electrode footprint represents less overlapping in the recorded signal. Consequently, the characterization of EII should also consider the contact area. An impedance normalized according to area (kΩ cm^2^) can better reflect the electrode performance [9, 33]. For long-term applications, flexible microneedle electrodes fitting a curved body surface provide a better tolerance to patient activity. Conversely, electrodes with rigid substrates may degrade or even fail due to relative sliding against the skin, and cause indentation or pain [13]. Therefore, while ensuring the strength of microneedles, more flexibility of the substrate is preferable. Additionally, manufacturing cost and efficiency are also considerations, as microneedle electrodes should be designed as disposable to avoid cross-infection. Photolithography and etching based on microfabrication technology are suitable for realizing microneedle structures [21, 29, 30, 34–36], but the expensive manufacturing equipment and environment limit wider promotion. New convenient approaches have been more commonly used to fabricate microneedles [33, 37–45]. However, although the fabrication methods and applications of MNAs have been widely researched, few works have reported on the application of biopotential dry electrodes with excellent performance. Thus, the proper design of the microneedle electrode will realize wearability, good electrode–skin contact, reduced manufacturing cost, and long-term availability for surface electrophysiological monitoring. In addition, the clinical application of microneedle dry electrodes has not yet been reported.

This paper reports a novel surface biopotential dry electrode based on a polyimide microneedle array (PI-MNA) that achieves high-quality electrophysiological recording. The novelty and advances of our work mainly lie in the following: 1) Excellent electrode–skin contact, long-term wearability, biosafety, and low cost. 2) This is the first report on the clinical study of microneedle electrodes for surface electrophysiological monitoring. The PI-MNA electrodes are applied to clinical long-term continuous biopotential recording for sleep monitoring combined with standard PSG instruments. 3) Compatibility with wearable wireless systems and short preparation times. A series of specialized wearable wireless recording systems for high SNR and low motion artifact biopotential recording combined with the PI-MNA electrodes.

## Materials and Methods

### Materials

Silicone elastomer PDMS (SYLGARD™ 184) was obtained from the Dow Chemical Company. The nonphotosensitive polyimide precursor (solid content of 14%) was acquired from POME Sci-Tech Co., Ltd. EDOT (97%, the monomer of PEDOT) and PSS (analytical standard, molecular weight of ~ 70,000) were purchased from Sigma-Aldrich LLC.

### Device Fabrication

The flexible microneedle array structure of the PI-MNA electrodes was mainly fabricated by means of a micromolding process. The manufacturing flow began with assembling the rigid master mold consisting of a drilled fiberglass board and machined Ni-plated steel needles. The aperture of the holes on fiberglass board and the diameter of steel needles were both designed as 200 μm, which defined the base diameter of the microneedles. The length of the steel needles and the thickness of the fiberglass board were 3 and 2.6 mm, respectively, and the length of the needle tips was machined to 0.4 mm. Thus, when steel needles were assembled into the hole array on the fiberglass board, conical microneedles with a length of 400 μm and a base diameter of 200 μm were exposed on the surface of the board. The back of the master mold was encapsulated with epoxy adhesive to fix the steel needles assembled into the holes.

The steel needles were manufactured using 0.2-mm-diameter music steel wire for higher mechanical strength. The 0.4-mm conical tip and 3-mm length were fabricated in sequence through standard machining processes including alignment, grinding, and wire cutting. A layer of ~ 2 μm Ni was electroplated on the surface of the steel needle by a rolling plating process to prevent rust. The fabrication of steel needles was provided by a general manufacturer qualified to produce medical devices such as puncture needles, injection needles, and acupuncture needles.

The finished master mold was placed in a Petri dish and poured into silicone elastomer PDMS. The ratio of the main and curing agents for PDMS was set as 12:1 to produce a soft negative mold. After 10 min of vacuuming to completely fill the small corners of the master mold, the PDMS was treated on a hot plate at 80 °C to solidify. Then, the cured PDMS was easily demolded to obtain a negative mold with a conical micropore array. Next, 0.1 g of low-viscosity, nonphotosensitive polyimide precursor was cast on the surface of the negative mold and vacuumed for 5 min to fill the micropores. After 1 h of leveling, the casted negative mold was placed on a hot plate at 120 °C to completely evaporate the solvent, followed by the imidization reaction at the 240 °C for 2 h. At this point, the polyimide film was demolded from the negative mold to obtain a flexible polyimide MNA. The thickness of the polyimide substrate prepared by the process can be less than 50 μm, ensuring good flexibility.

Magnetron sputtering was employed to deposit conductive metal layers on polyimide MNA. Ti (10 nm) and Au (200 nm) were used as the adhesion layer and low-resistance conductive layer, respectively. Here, sputtering was separately performed on the front and back of the polyimide MNA, so the Ti/Au layer could cover every surface including the sidewall of the flexible substrate. The back was electrically conductive to the microneedles. In subsequent impedance and recording tests, the leads were glued to the back of the PI-MNA electrodes by conductive silver adhesive to transmit the signal.

### Mechanical Tests

The penetration force and strength conformance tests of the PI-MNA electrodes were performed using standard mechanical testing equipment (1ST, Tinius Olsen Co., Ltd.). As the pressure probe moved, the displacement and contact force were recorded simultaneously. For the skin penetration test, a fresh piece of pig skin was used as an object for microneedle piercing. The PI-MNA electrode was stuck on the probe that moved uniformly downward to the pig skin. The stop point of the test was set at 5 N, and a transient drop in pressure was observed during the test, representing the moment when the MNA penetrated the skin. For the strength conformance test, the PI-MNA electrode was placed on the test platform with the microneedles facing up, and the probe moved downward to directly apply pressure to the MNA. The stop point was set to 500 N, and the compression distance of the probe itself was corrected by software.

### Surface Modification

The conducting polymer PEDOT/PSS was modified on the gold surface of the PI-MNA electrode by a galvanostatic electrodeposition approach in a two-electrode system. Monomer EDOT (0.01 M; Sigma-Aldrich) and PSS (2.5 wt%; Sigma-Aldrich) were dissolved in deionized water and thoroughly mixed. An electrochemical workstation (CHI 660E, CH Instruments, Inc.) was employed to apply a small but steady deposition current. The PI-MNA electrode was connected to the working electrode of the electrochemical workstation through an electrode clamp, and a gauze Pt electrode was connected to counter and reference electrode. After both electrodes were completely immersed in the EDOT and PSS solution, the deposition current was applied, and PSS-doped PEDOT was polymerized on the surface of the working electrode. It was considered that an excessive deposition current would induce a polymerization speed that is too fast, thus forming a rough, fluffy PEDOT/PSS film that easily cracks or chips on the gold surface. A step-up current was used to enhance the adhesion of PEDOT/PSS. Deposition currents of 20, 40, 60, and 80 μA were each applied for 150 s, followed by 100 μA for 600 s. The thickness of the deposited PEDOT/PSS film was approximately 1 μm.

### Impedance Characterization

The EII spectra of the electrodes were measured by a two-electrode system of electrochemical workstation (CHI 660E). During the tests, the PI-MNA electrodes were attached to the volunteer’s skin by medical tape and were connected to the instrument from the back by leading wires bonded with conductive silver adhesive. A volunteer (24-year-old male) was required to wear pairs of electrodes attached to the electrochemical workstation on the forearm. The distance between the two electrodes was 5 cm. The initial and stimulus voltages of the EII test were set as 0.7 and 0.3 V, respectively. Here, the tests yielded the addition of two EII impedances. To fully compare the difference in contact impedance between different electrodes, four types of electrodes, including the PI-MNA electrodes with PEDOT/PSS and Au surface, clinical standard wet electrodes (gel electrode: 3 M 2223CN; goldcup electrode: Tenocom Medical Technology Co., Ltd.; Fig. S3a-b), were each used 20 pairs for frequency sweep in the range of 0.1 Hz to 1 MHz. The skin surface to attach the electrodes was cleaned and disinfected by wiping with 75% alcohol prior to the tests. Considering that different types of electrodes have different contact areas with skin, the measured EII values were normalized as follows:

1 $$\left| Z \right|_{{{\text{normalized}}}} = \left| Z \right|_{{{\text{measured}}}} \times {\text{Area}}_{{{\text{contact}}}}$$

The contact area of the PI-MNA electrode was counted as the actual footprint, *i.e.*, 0.36 cm^2^, the contact area of the gel electrode was counted as only the gel area (2.25 cm^2^) rather than the footprint of the larger medical adhesive, and the contact area of the goldcup electrode was as large as the round footprint of the “cup” (0.785 cm^2^). In the actual use of the goldcup electrode, the conductive gel would overflow the outer diameter of the cup, so the actual contact area would be larger than 0.785 cm^2^. The data shown in Fig. 4d, e are the mean values ± standard errors from 20 pairs of each electrode type.

### Wireless Recording System Development

Three types of wearable wireless recording systems based on FPC were built to acquire ECG, EMG, EOG, and EEG signals in real time. The first system was developed for ECG and EMG recording (Fig. S7a). The FPC is divided into three parts with a total area of approximately 6.5 × 3 cm^2^. The three parts are connected by serpentine stretchable lines to accommodate skin deformation that may be caused by user movement. Three PI-MNA electrodes can be fixed by conductive adhesive at the connection points located at the back of the electronic system as differential input pairs of the front-end amplifier and a right leg drive (RLD) electrode that can significantly suppress the input noise. The distance between the two adjacent electrodes is 2.5 cm. The right part is arranged with a power management circuit that mainly contained a low dropout regulator (LDO) chip (TLV71333, Texas Instruments, Inc.). A 3.7 V rechargeable lithium-ion battery is also placed here and soldered to the battery PADs. The middle part is the application specific integrated circuit (ASIC) chip (AD8232, Analog Devices, Inc.) for ECG recording and its peripheral circuit. The signals collected by the electrode is amplified and filtered here. The gain and frequency band are set to 600 and 0.5–40 Hz, respectively. The left part is the microcontroller unit (MCU) chip (nRF52832, Nordic Semiconductor ASA) with a 12-bit analog-to-digital converter (ADC) and a Bluetooth low-energy (BLE) module. Analog signal processed from the front-end is converted to digital signals with the sampling rate of 1 kHz and transmitted wirelessly.

The second system was developed for EOG recording (Fig. S7b). The circuit composition of the EOG recording system is the same as that of the ECG/EMG system, except for the shape of the FPC, the layout of the electronic components, and the position of the electrodes for better fitting around the eye. Two differential input electrodes are worn near the inner and outer canthus, and the RLD electrode is worn behind the ear.

The third system was developed for EEG recording (Fig. S7c). The shape of the FPC is designed to wear the forehead and softly connect the mastoids behind the ears. Two input electrodes are located on both sides of the forehead, differentially with the reference electrode at the right mastoid, and the ground electrode is at the left mastoid. The circuits located in the middle of the forehead contained the ASIC chip (ADS1299, Texas Instruments, Inc.) for EEG recording, the MCU chip (nRF52840, Nordic Semiconductor ASA), the LDO chip (TLV70233, Texas Instruments, Inc.), and the 3.7 V rechargeable lithium-ion battery. Two of the recording channels of ADS1299 were employed to acquire frontal EEG signals from symmetrical positions. The gain and frequency band of the front-end integrated in ADS1299 were set to 24 dB and 1–50 Hz, respectively, and the processed signal was converted by the 24-bit built-in ADC. Digital data were input to nRF52840 and wirelessly transmitted via BLE.

Another PCB contained MCU chip nRF52832 and connected to a computer through USB served to receive the signals and input them to the computer through a serial port. A graphical user interface (GUI) software based on MATLAB was used for real-time display and storage of the data.

### Motion Artifact Characterization

The ECG from the chest was used to characterize the artifacts of the PI-MNA electrode and gel electrode during electrophysiological signal recording. Seven kinds of active states, including resting (static standing), light exercise (slow walking, fast walking, jogging), vigorous exercise (running on spot), and special breathing (deep breathing, rapid breathing), were performed. Signals from two electrode types were recorded for 15 s at the same time in each of the states. Three data analysis methods were used to quantify the artifacts present in ECG signals. First, the SD was calculated for all data points of each ECG signal to indicate the overall divergence of the signal. In addition, peak values of the QRS complex can also reflect the artifacts. In the case of no artifact, the QRS peaks should be consistent. The difference between the maximum and minimum values, as well as the SD of QRS peaks in each ECG signal, were calculated. Larger differences and SD values represented the more obvious artifacts.

### SNR Analysis

EMG signals were recorded to evaluate the SNR performance of the PI-MNA electrodes with the wearable wireless recording system. Three limb muscles, the extensor digitorum, biceps, and tibialis anterior, located in the forearm, upper arm, and lower leg, respectively, were included in the test. The volunteer was required to repeat ten actions, and the EMG signals were recorded for 20 s. For the extensor digitorum, contraction and wrist lifting were carried out. For biceps, the actions included contraction and elbow bending separately at slow and fast rates. For the tibialis anterior, five motions of contraction, ankle lifting, walking, knee bending, and leg lifting were considered. Low-frequency motion artifacts on the recorded raw data were filtered to obtain flat baselines for SNR analysis via a 20 Hz high-pass finite impulse response (FIR) digital filter developed by MATLAB. Meanwhile, a 50-Hz harmonic infinite impulse response (IIR) comb digital filter was employed to eliminate the power frequency interference. The signal and noise segments were extracted from the filtered data. In the 20-s recorded data, 300-ms data from the middle of each high-amplitude EMG waveform triggered by muscle activities were taken as the signal segments, and 600-ms data from the middle of the relaxing part between each two EMG waveforms were taken as the noise segments. The RMS values of the signal and noise data of all extracted segments from each EMG signal were calculated to reflect the average amplitude. The RMS value was calculated as follows:

2 $${\text{RMS}} = \sqrt{\frac{1}{N}} \mathop \sum \limits_{n = 1}^{N} \left| {X_{n} } \right|^{2}$$

where *N* is the total number of data points and *X*_*n*_ is the *n*th data point. The SNR was calculated as follows:

3 $${\text{SNR}} = 20{\text{log}}_{10} \frac{{{\text{RMS}}_{{{\text{signal}}}} }}{{{\text{RMS}}_{{{\text{noise}}}} }}$$

Furthermore, the SNR difference between the PI-MNA and gel electrodes was characterized by EMG channels of a PSG instrument, Compumedics Grael [53]. To minimize the impact of different electrode-instrument connections, in this test, the PI-MNA electrodes were bonded by conductive adhesive to gel electrodes, which were stripped off the gel with the Ag/AgCl plate exposed, and connected to the instrument through standard button connectors. Similarly, EMG signals were separately recorded from the extensor digitorum, tibialis anterior, and biceps. A volunteer (22-year-old male) wore pairs of PI-MNA electrodes and pairs of gel electrodes over the same muscle, and the “left” and “right” recording sites were defined in the direction of the first view. The distance between the two electrodes on the same side was set to 5 cm. EMG signals during muscle contraction were recorded simultaneously, and then electrode positions were switched to record again. During each EMG recording time, the muscle alternately contracted and relaxed for 12 s, and this process was repeated 5 times. The 10-s data from the middle of the contacting and relaxing parts were taken as the signal and noise segments, respectively. Five signal segments and five noise segments were obtained from the EMG data under each condition. RMS and SNR were calculated from each pair of segments. The mean values and standard errors were statistically analyzed.

### PSG Data Recording and Analysis

To demonstrate the applicability of the PI-MNA electrode for PSG, a volunteer (28-year-old male) took sleep monitoring for two nights using a standard PSG instrument, Compumedics E-series [53]. Electrophysiological signals, including EEG from the frontal, parietal, and occipital regions (reference to the contralateral mastoid), EOG from the left and right eyes (reference to the contralateral mastoid), chin EMG, ECG, and bilateral leg EMG from the tibialis anterior, were continuously recorded during sleep (Fig. S9). For the first night, F3, LOC, A2, Chin1, ECG1, Lleg1, and Lleg2 were connected to the PI-MNA electrodes. Other sites were connected to wet electrodes (goldcup electrode: REF, GND, F4, C3, C4, O1, O2, A1, Chin2; gel electrode: ECG2, Rleg1, Rleg2). For the second night, almost all employed electrodes were PI-MNA electrodes, except hair-covered sites C3, C4, O1, and O2. The PI-MNA electrodes were attached to skin with medical tape and were connected to the instrument from the back. Overnight data recorded by the PSG instrument were stored in EDF format and read by EEGLAB [54]. Typical EEG and EOG of different sleep stages, including K-complex wave, spindle wave, δ wave, and rapid eye movement, were selected from the data, and the Pearson correlation coefficients as well as P values of the data synchronously recorded at symmetrical sites were calculated. Moreover, the α wave, α wave block, horizontal eye movement, reading eye movement, chin EMG, and leg EMG were recorded during waking time, when the volunteer was required to keep eyes closed, keep eyes open, move eyes horizontally, read a passage, grit teeth, and lift an ankle. The power spectrum density (PSD) of the typical EEG waveforms was obtained by fast Fourier transform (FFT) via MATLAB.

#### Clinical Study Protocol

The clinical study of the PI-MNA electrodes for PSG was conducted in two groups, the first group enrolled healthy subjects (five males and seven females from 21 to 29 years old), and the second group enrolled subjects with OSAHS symptoms (15 males and one female from 22 to 37 years old). All healthy subjects were judged as having good sleep quality, and the other group of subjects were screened as having high risks of OSAHS by standard questionnaires. After each night of sleep monitoring, the subjects were required to fill out another questionnaire about the comfort of PSG, including the comfort during awakening, during sleep, and of the instrument (Supplementary File S1).

Three types of PSG instruments were employed and more than 7 h of monitoring were guaranteed for each night. For healthy subjects, five used Compumedics Grael and seven used Compumedics E-series. The subjects were required to participate for one night of monitoring. Recording sites F3, LOC, A2, Chin1, Chin2 (E-series), ECG1, ECG2, Lleg1, and Lleg2 were connected to the PI-MNA electrodes. Other sites were connected to wet electrodes (goldcup electrode: F4, C3, C4, O1, O2, A1, Chin2 (Grael), Chin3 (Grael), REF, GND; gel electrode: Rleg1, Rleg2). For OSAHS subjects, nine used Compumedics E-series and seven used the portable instrument Compumedics Somté [53]. The subjects were required to participate for two nights of monitoring. On the first night, all sites were wet electrodes. On the second night, almost all sites were PI-MNA electrodes except the hair-covered C3, C4, O1, O2 sites and leg EMG sites of Somté with special wires. Adequate skin preparation was carried out on the positions of wet electrodes, ensuring EII < 10 kΩ at 1 kHz for EEG, EOG, and chin EMG sites, as well as EII < 20 kΩ at 1 kHz for ECG and leg EMG sites. Before the beginning of every night’s monitoring and at the end in the morning, the 1 kHz EII of all electrodes measured by the PSG instrument were recorded.

A total of 44 nights of PSG data were provided to an experienced physician who had been approved as Registered Polysomnographic Technologist (RPGST) for sleep staging. For 12 nights of data from healthy subjects, the physician was required to stage three times, including all channels (full-lead), F3-only for EEG and LOC-only for EOG (the PI-MNA single-lead), F4-only for EEG and ROC-only for EOG (wet electrode single-lead). The clinical sleep physicians were blinded to group allocation during clinical PSG data collection and analysis, the data were disordered, and the subjects’ numbers were hidden. Eleven indicators extracted from the staging results were included in the statistical analysis of different staging methods, and paired-samples t tests were performed to characterize the correlations. For 32 nights of data from OSAHS subjects, the statistical analysis was conducted mainly on sleep efficiency from sleep staging results and subjective comfort from questionnaires.

## Results

### Structure, Materials and Manufacturing Approach

**Fig. 1:**
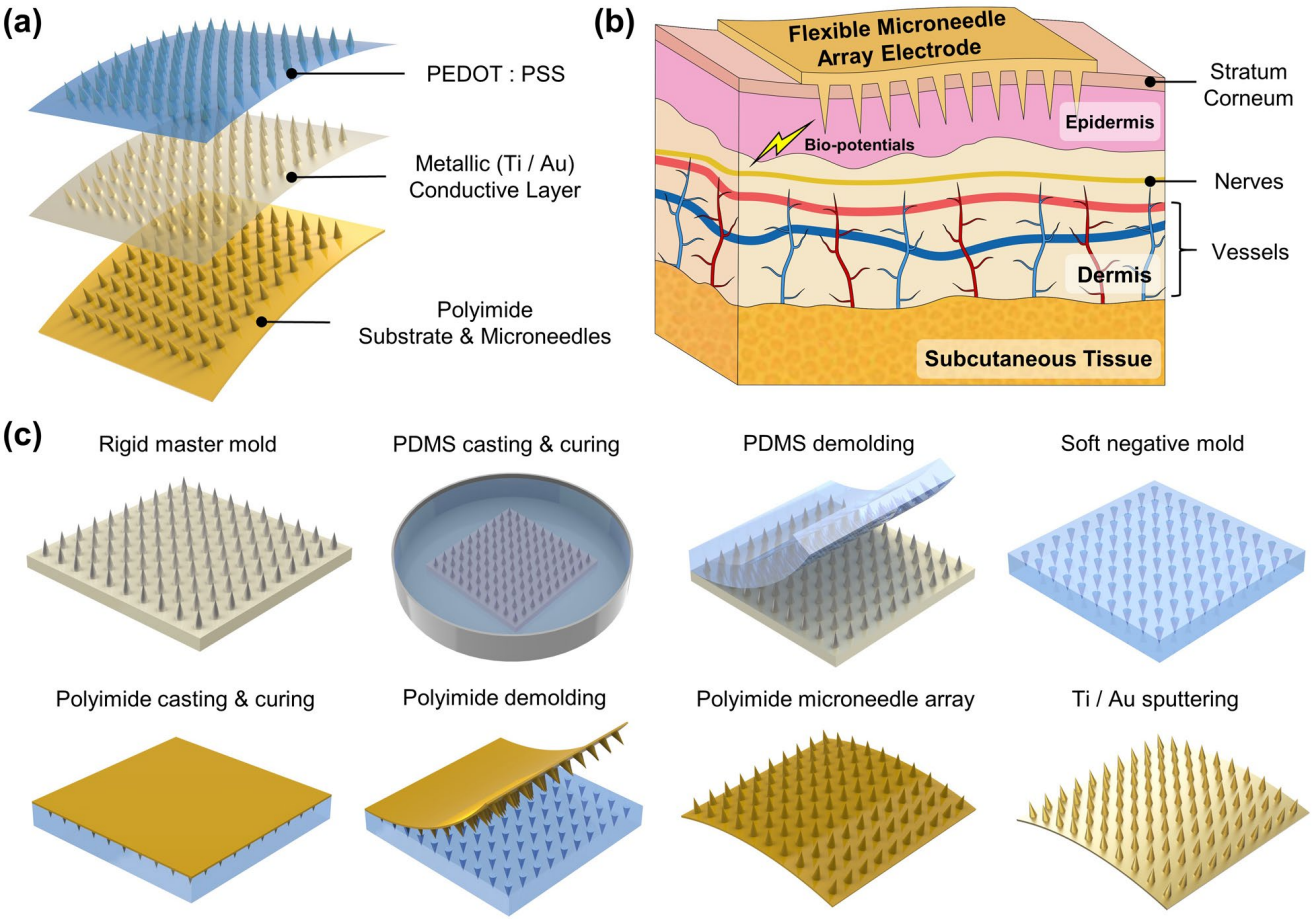
**a** Schematic view of the structure and material of the PI-MNA electrode. **b** Principle of ultraminimally invasive biopotential acquisition by a flexible MNA electrode. **c** Manufacturing process flow of the PI-MNA electrode

Figure 1a illustrates the basic structure of the PI-MNA electrode. One hundred microneedles are arranged as a 10 × 10 array on a substrate 6 × 6 mm^2^ in size. The base diameter, length, and pitch of the microneedles are set as 200, 400, and 500 μm, respectively. Both the MNA and flexible substrate are made of polyimide and have an integral structure. Polyimide, a polymer with excellent chemical and thermal stability, has been extensively demonstrated in biomedical applications both in vitro and in vivo as a flexible, insulating and passivation material [46]. Two layers of conductive film are deposited on all surfaces of the device, including the microneedles, the sidewalls, and the back of the substrate. Metal layers of titanium (Ti), gold (Au) and conducting polymer poly(3,4-ethylenedioxythiophene)/poly(styrene sulfonate) (PEDOT/PSS) are aimed at adhesion, signal transmission, and enhancement of electrode–skin contact, respectively. Biopotentials acquired by the microneedles can be transmitted back through the fully covered conductive layers and further lead to external instruments.

As shown in Fig. 1b, when the flexible PI-MNA electrode is attached onto skin, the microneedles can effectively cross the SC and directly contact the epidermis without touching the dermis and deeper tissues where nerves and vessels exist [27]. Thus, highly efficient biopotentials acquisition is achieved by the PI-MNA electrode with ultraminimal invasion. The integrated microneedle and substrate ensure the robustness of the device. Compared to previously reported MNA devices with different substrate materials [29–32, 42, 44], the latter have higher risks of microneedles falling off during use. Meanwhile, compared to microneedles made of brittle materials such as silicon [29, 30, 34, 35] or SU-8 [43, 44], polyimide microneedles tend to bend rather than break when affected by external forces. Therefore, the structure and material design of the PI-MNA electrode can bring higher biosafety.

Figure 1c shows the manufacturing flow of the PI-MNA electrode. The polyimide substrate and microneedles are basically fabricated by micromolding that can conformally transfer the structure from an assembled master mold. Polydimethylsiloxane (PDMS) is employed as the intermediate negative mold. The Ti/Au layer is deposited by sputtering to ensure good step coverage and adhesion. The significant advantages of the above manufacturing process are its low cost and high efficiency, making it suitable for mass production. The cost of each PI-MNA electrode is estimated at $0.35, and thousands of electrodes can be manufactured per operator per day.

**Fig. 2:**
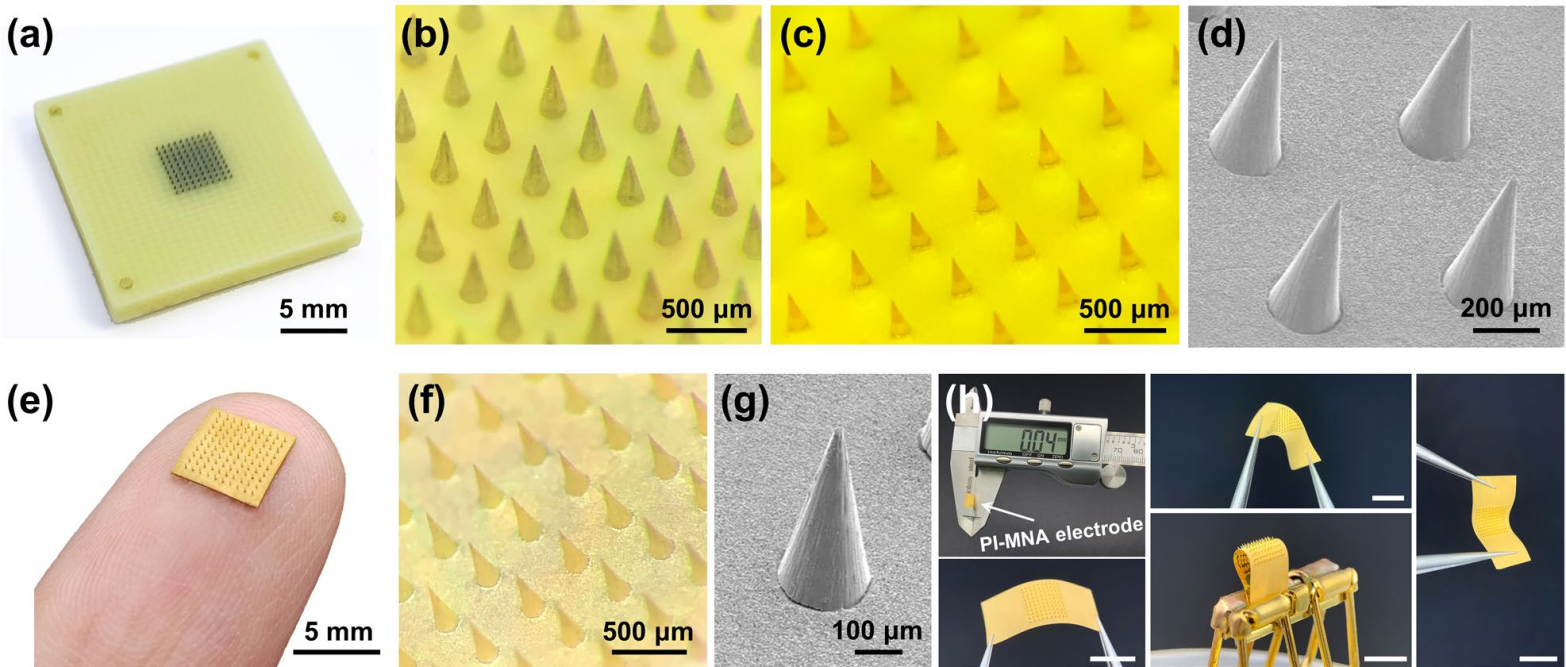
**a** Photograph and **b** micrograph of the assembled rigid master mold. **c** Optical and **d** SEM micrographs of polyimide microneedles after micromolding. **e** Photograph, **f** optical and **g** SEM micrographs of the PI-MNA electrode after Ti/Au deposition. **h** Thickness and distortion characterization of the PI-MNA electrodes (scale bar: 5 mm). For the convenience of the tests, redundant areas were reserved at opposite sides of the MNA

Figure 2a-g provides photographs and micrographs of the rigid mater mold, polyimide microneedles, and PI-MNA electrode after metal deposition, showing good reproduction during micromolding and dense coating of the metal layer on every tiny corner. The tip diameter of the polyimide microneedle was less than 10 μm, which is spiky enough to penetrate the skin effectively. As shown in Fig. 2h, the thickness of the polyimide substrate was controlled within 50 μm to ensure good flexibility.

### Mechanical Properties, Electrical Characteristics, and Biocompatibility

**Fig. 3:**
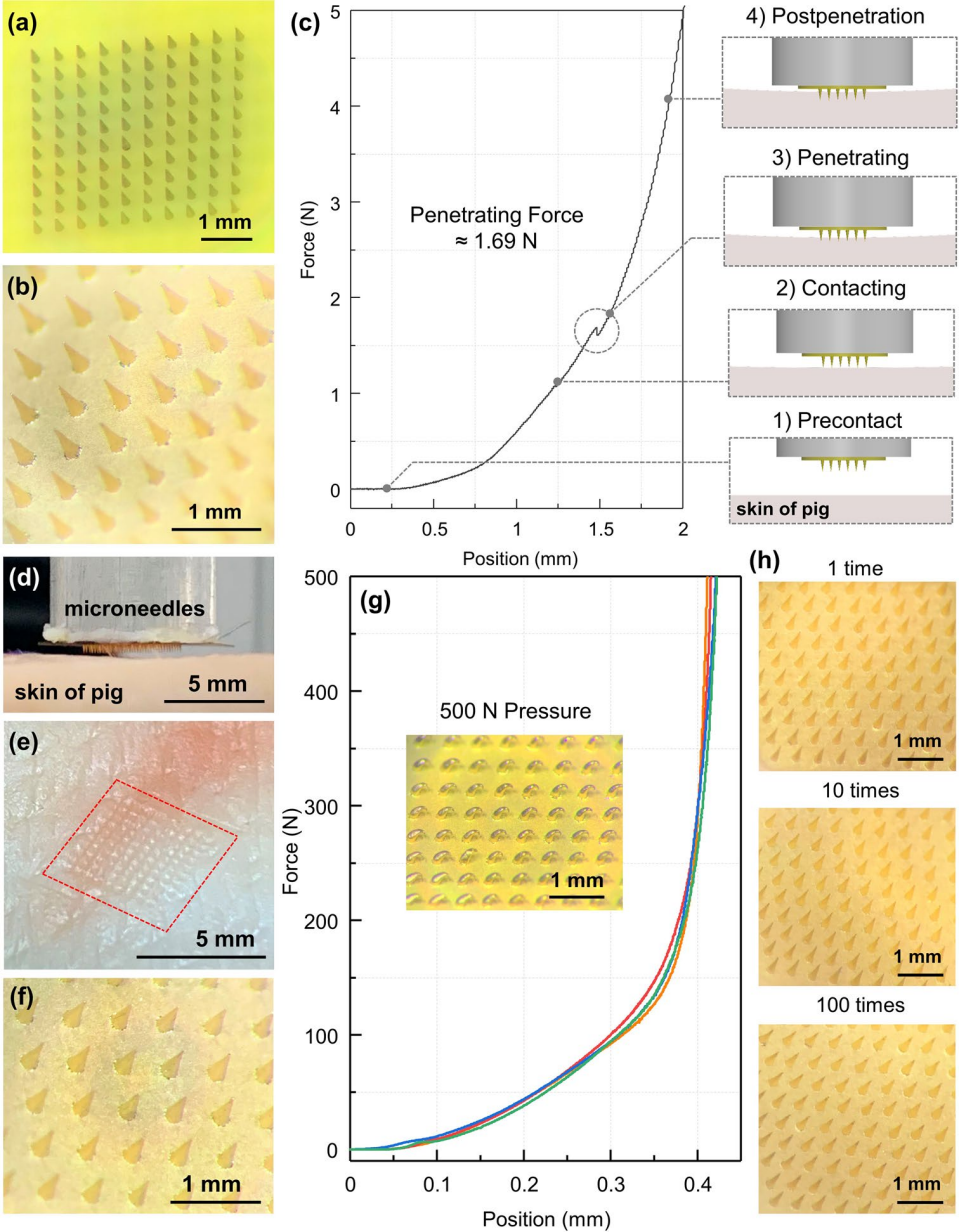
Micrographs of **a** the master mold after repeatedly micromolding for 25 times and **b** the PI-MNA electrode fabricated from the 25th micromolding of the same master mold. **c** Pressure–displacement curve tested during the PI-MNA electrode application to a piece of pig skin and the schematic diagrams when precontact, contacting, penetrating, and postpenetration. **d** Photographs of the penetration test. **e** Micrographs of the surface of pig skin after PI-MNA electrode penetration and **f** the surface of the PI-MNA electrode after penetrating the pig skin at 5 N pressure. **g** Pressure–displacement curves of force applied to the PI-MNA electrode up to 500 N through a rigid pressure probe (n = 4). Inset: micrograph of the microneedles after 500 N extrusion. **h** Micrographs of the PI-MNA electrode surface after multiple applications to human forearm skin

Reliable process repeatability ensures the good morphology of microneedles and controls manufacturing cost. To verify the robustness of the process, the same rigid master mold was repeatedly micromolded for 25 times. Figure 3a, b shows the master mold after 25 times of micromolding cycles and the PI-MNA electrode fabricated from the 25^th^ process. After repetition, the master mold surface was still clean and smooth, the steel needles showed no deformation, and the micromolded PI-MNA electrode morphology was still perfect. The master molds made of steel and fiberglass offered a strength and lifetime far superior to those of lithographic or 3D-printed molds [13].

To characterize the mechanical strength required for the PI-MNA electrode to penetrate skin, a pressure–displacement curve was tested on pig skin, as shown in Fig. 3c. The photograph in Fig. 3d shows the actual test configuration. When the pressure reached ~ 1.69 N, the MNA penetrated the skin surface and showed an instantaneous pressure reduction on the tested curve. The penetration holes on the pig skin were clearly visible (Fig. 3e), indicating that the microneedles effectively entered the subcutaneous area. The structure of the microneedles after penetration was still intact without obvious deformation (Fig. 3f). Furthermore, the steel pressure probe was used to apply force directly to the PI-MNA electrodes up to 500 N. As shown in Fig. 3g, similar curves were obtained from several tests on different electrodes, showing that the mechanical properties were in good consistency. The inset in Fig. 3g shows the microneedles after extrusion of 500 N, which is much higher than the pressure required for skin penetration. The microneedles were almost completely bent but still not broken, demonstrating the biosafety benefiting from the flexibility of the polyimide. To validate the safety in practical applications to the human body, the PI-MNA electrodes were applied to human forearm skin 100 times (volunteer: 26-year-old male). As shown in Fig. 3h, the morphology of the MNA hardly changed after one use. With the increase in application times, the microneedle tips degraded slightly, but no fracture occurred. In actual use, the PI-MNA electrodes were disposable taking advantage of their very low cost thus effectively avoiding safety risks.

**Fig. 4:**
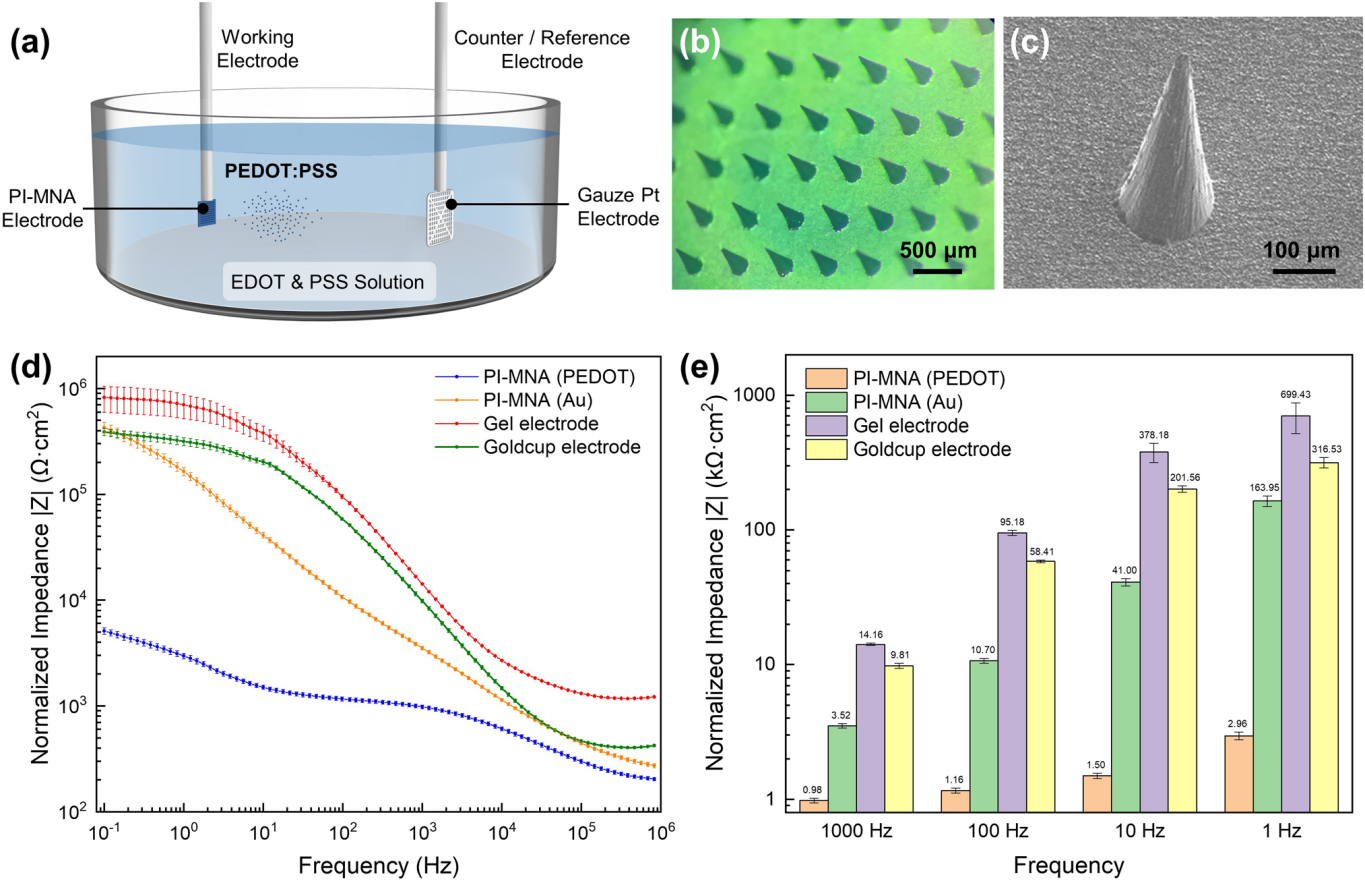
**a** Schematic view of the electrochemical polymerization approach of PEDOT/PSS modification. **b** Optical and **c** SEM micrographs of the PI-MNA electrode after PEDOT/PSS modification. **d** EII spectra of different electrodes in the frequency range from 0.1 Hz to 1 MHz (*n* = 20, mean ± standard error). **e** The specific EII modulus values (*n* = 20, mean ± standard error)

Owing to the microneedles that can cross the highly resistive SC, the EII of the PI-MNA electrode is mainly due to the coupling capacitance, which is formed by the double electric layer at the ion–electron conversion interface. For MNA electrodes with metal surfaces, the conversion interface is located between the microneedles and subcutaneous tissue fluid, which results in a relatively high EII. Modifying conducting polymers on metal electrodes is an effective method to eliminate the double electric layer capacitance and reduce contact impedance [47]. Conducting polymers are able to exchange ions with surrounding electrolytes, thus showing ionic-electronic mixed conduction, which is essential to transduce biological ionic signals into electronic ones [48]. The most reliable conducting polymer for biomedical applications is PEDOT/PSS due to its high conductivity, good electrochemical stability, low intrinsic cytotoxicity or antigenicity, and simple deposition approach [46–51]. As shown in Fig. 4a, PEDOT/PSS was deposited on the PI-MNA electrode by electrochemical polymerization to completely shield the Au surface. The micrographs in Fig. 4b, c shows that PEDOT/PSS covered the electrode surface uniformly and compactly.

Figure 4d, e displays the EII of different electrodes on human skin. Clinical standard electrodes (gel electrode and goldcup electrode, Fig. S3a, b) were employed for comparison. The measured impedance was normalized according to the effective contact area of the electrodes. The results show that the EII of the PI-MNA electrodes with the Au surface was better than that of the wet electrodes, while the EII of the PI-MNA electrodes with the PEDOT/PSS surface was much superior to that of the other three types, especially in the low-frequency range (< 1 kHz) determined by surface biopotential recording. The impedance modules were reduced by approximately 15, 80, 250, and 235 times at 1000, 100, 10, and 1 Hz, respectively. Meanwhile, the EII level of the PI-MNA electrodes shown here is the record low value compared to previous reports [4, 5, 7–10, 29–32, 34, 41].

One of the significant advantages of dry electrodes is that they are more suitable for long-term applications, due to needlessness of conductive gel and better comfort. To verify the long-term stability of the PI-MNA electrodes, two special EII tests were carried out and proved the long-term performance and the contact properties in the sweating state: (1) the PI-MNA electrodes were worn on the volunteer’s skin for 48 continuous hours, and EII was measured every 12 h to characterize the long-term performance; (2) the volunteer was required to run outdoors for 30 min, while wearing the PI-MNA electrodes and the EII was separately measured before and after running to demonstrate the contact properties in the sweating state. The results (Fig. S4a, b) show that the EII remained stable in both cases, regardless of whether the electrode surface was modified. Moreover, the EII was further lower after exercise due to the lower resistance of sweaty skin (Fig. S4b).

Additionally, to evaluate the stability in electrical characteristics due to bending, EIIs were measured when the PI-MNA electrodes were attached to significantly curved skin surfaces. Tests were performed in two cases: (1) a pair of PI-MNA electrodes attached to the back of the wrist when the wrist was bent for 90° and 2) two PI-MNA electrodes attached to knuckles of the index and middle fingers of the same hand when the knuckles were bent for 90°. As shown in Fig. S4c, EII in bent states rose slightly compared to a pair of electrodes attached to flatter forearm skin, but remained at a very low level, enabling high-quality signal recording. The results verified the advantages of the PI-MNA as a flexible electrode.

According to the widely recognized biocompatibility of the materials, the PI-MNA electrodes should not cause any inflammation or allergic reactions when worn on skin in an ultraminimally invasive manner. As a verification, the PI-MNA electrodes were continuously worn on human skin for 2 h (volunteer: 26-year-old male), and the skin reaction was observed at the wearing positions. Figure S5 shows that the marks of microneedles could completely disappear within 1 h after removal, while the indentation of the gel electrode was still slightly visible, proving that the PI-MNA electrodes have good biocompatibility and biosafety. In addition, to further demonstrate the safety of the PI-MNA electrodes, energy dispersive spectroscopy (EDS) analysis was employed to prove that there was no surface material residue during the insertion and retraction of the PEDOT/PSS-modified microneedles. As shown in Supplementary Fig. S6, a piece of agarose gel was used as the insertion substrate, and the microhole array can be clearly seen. EDS analysis in and out of the insertion hole showed that the element composition was consistent. No sulfur, the characteristic element of PEDOT/PSS, was detected, indicating that there was no residual material after applying the PI-MNA electrode.

To date, considering all the experiments that have been conducted, thousands of PI-MNA electrodes have been applied to the human body without causing inflammation, allergies or other adverse reactions. Microneedles caused only a slight prickling sensation during the moment of skin contact, and the penetration marks usually spontaneously faded within hours.

### Wearable Biopotentials Recording via Wireless Flexible Electronics

The most important application of PI-MNA electrodes is wearable biopotential recording. The PI-MNA electrodes can be easily and directly worn on the body surface without interfering with daily activities, because the contact area is significantly smaller than that of wet electrodes and the conductive gel is not needed. A recording system we developed based on flexible printed circuit (FPC) board with stretchable serpentine structure (Fig. S7a) can be worn at the ECG and EMG acquisition positions. The biopotentials collected by the electrodes were processed in situ and wirelessly transmitted.

**Fig. 5:**
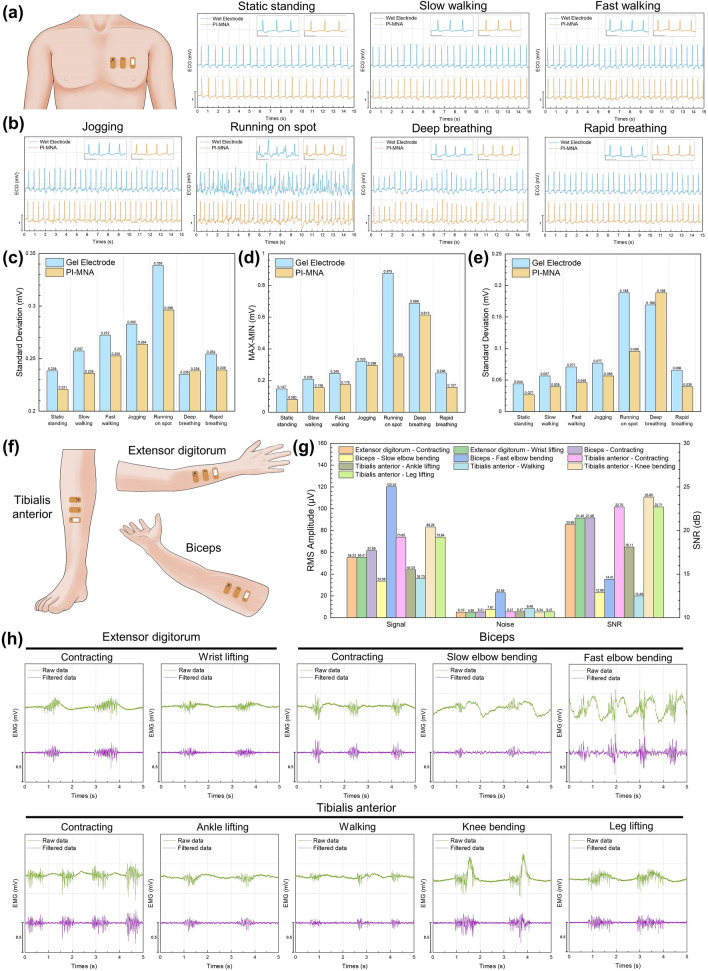
**a** Wearing position for ECG wireless recording. **b** Simultaneous ECG signals recorded during seven different states by the wearable system with the PI-MNA and gel electrodes. The insets in each diagram show the clearer signal segments to visually display the artifacts (scale bar: x—0.5 s, y—0.5 mV). **c-e** Quantitative analysis of the motion artifact level of ECG, including** c** the standard deviation of all data points, **d** the difference between the maximum and minimum peak values of the QRS complex, and **e** the standard deviation of the QRS peaks. **f** Wearing position for EMG wireless recording. **g** RMS values of signals and noises during recording, as well as the SNR values of EMG signals in 10 different muscle or joint movements. **h** 5-s segments of EMG recording

Conventional wet electrodes contact skin through conductive gel. When users move or breathe, the contact interface tends to slide, which reduces the recognition of signals due to artifacts. The artifacts have a great influence on the diagnosis of cardiac diseases requiring Holter. For patients wearing ECG electrodes for a whole day, movements are inevitable, and artifacts can cause distortion or even unreadable waveforms in part of the time. Previous reports have noted that flexible microneedles could penetrate the skin and maintain a more stable contact interface, weakening the effect of motion artifacts [25]. To validate the anti-artifact capability of the PI-MNA electrodes and wearable recording system, ECG signals were simultaneously recorded by the system with the PI-MNA and gel electrodes at the left chest of the human body (Fig. 5a). A volunteer (22-year-old male) was required to simultaneously wear two ECG/EMG wireless recording systems connected with the PI-MNA electrodes and gel electrodes. A transparent, waterproof, stretchable patch (3 M Tegaderm) fully covered the wearable recording systems to stabilize the recording system. As shown in Fig. 5b, seven active states were considered. Figure 5c−e provides three quantitative analysis methods to characterize the artifact level of the recorded ECG, including the standard deviation (SD) of all data points, the difference between the maximum and minimum peak values of the QRS complex, and the SD of the QRS peaks. All three methods show that the ability of the PI-MNA electrodes to suppress artifacts is generally superior to that of wet electrodes.

The same wearable recording system can be applied to acquire EMG signals. The extensor digitorum, biceps, and tibialis anterior (Fig. 5f) were chosen to characterize EMG recording performance. The ECG/EMG wireless recording system connected with the PI-MNA electrodes was worn on skin over the muscles of the volunteer, and stabilized by an adhesive patch (3 M Tegaderm). Wearing the PI-MNA electrodes and the recording system, ten types of activities including contraction and relative movements were performed. As shown in Fig. 5g, the root-mean-square (RMS) values of signals and noises during recording, as well as the SNR of EMG signals for each active state were calculated. Figure 5h provides the 5-s segments of EMG recording. Because the muscle activities were relatively intense, the raw data still showed obvious artifacts, and digital filtering methods were carried out to improve signal quality. The calculated values in Fig. 5g were derived from the 20-s filtered data (Fig. S8). EMG signals recorded in most of the active states can maintain similar noise levels (< 10 μV). A special case was the EMG when rapid elbow bending occurred. Because the high-amplitude EMG signal appeared at short intervals, and the muscles were not fully relaxed between two adjacent elbow bending activities, part of the low-amplitude EMG signal was counted as noise, resulting in a higher noise level and lower SNR. The SNR differences of other cases mainly depended on the amplitudes of EMG signals.

**Fig. 6:**
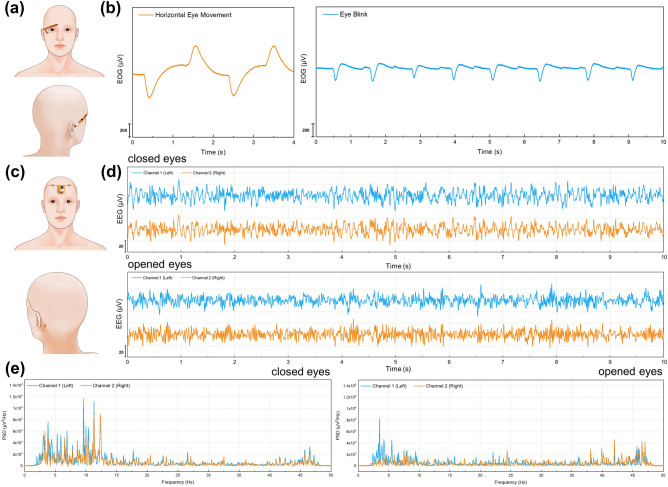
**a** Wearing position for EOG wireless recording. **b** EOG waveforms during a horizontal eye movement and eye blink. **c** Wearing position for EEG wireless recording. **d** Two-channel frontal EEG waveforms when eyes were closed and opened. **e** Frequency analysis of frontal EEG (PSD: power spectral density)

Based on the same circuit design as the ECG/EMG wireless recording system, the FPC layout was redesigned for EOG acquisition (Fig. S7b). The EOG recording system with the PI-MNA electrodes was used to acquire signals during eye movement and blink. As shown in Fig. 6a, the wireless system was worn around the right eye orbit of the volunteer so that two differential input electrodes were located near the inner and outer canthus, and the RLD electrode was located next to the right ear. The EOG waveforms showed good and stable signal characteristics (Fig. 6b). Furthermore, a two-channel wireless frontal EEG recording system was also developed (Fig. S7c). As shown in Fig. 6c, the wireless system was worn on the middle of the forehead with two input electrodes placed at the F3 and F4 sites (defined by the international 10–20 system). The reference electrode and ground electrode were placed at the mastoid behind the right and left ear, respectively. Two-channel EEG signals in the resting state were recorded, showing α wave characteristics and α wave block phenomena when eyes were closed and opened, respectively (Fig. 6d, e). Here, to keep the EOG/EEG wireless recording systems and the PI-MNA electrodes firmly adhered to the skin, the FPC was affixed with medical double-sided adhesive in the free area between the electrodes, and further secured with medical adhesive on the back of the FPC around the electrodes.

### Long-term Continuous Biopotential Recording for Polysomnography

PSG is a typical application of long-term biopotential recording that can monitor sleep status overnight and classify different sleep stages. PSG can be used to diagnose dozens of sleep disorders, and is the gold standard for obstructive sleep apnea hypopnea syndrome (OSAHS) diagnosis [52]. Sleep staging based on biopotentials is the key to interpreting sleep. At present, all electrodes used in clinical PSG are wet electrodes, of which goldcup and gel electrodes are the most commonly used. The liquidity of the conductive gel necessary for goldcup electrodes, the large sticking area of gel electrodes, and the tedious procedure of skin preparation can cause patient discomfort. In this work, we replaced the wet electrodes attached to PSG instruments with PI-MNA electrodes for electrophysiological recording and sleep monitoring.

**Fig. 7:**
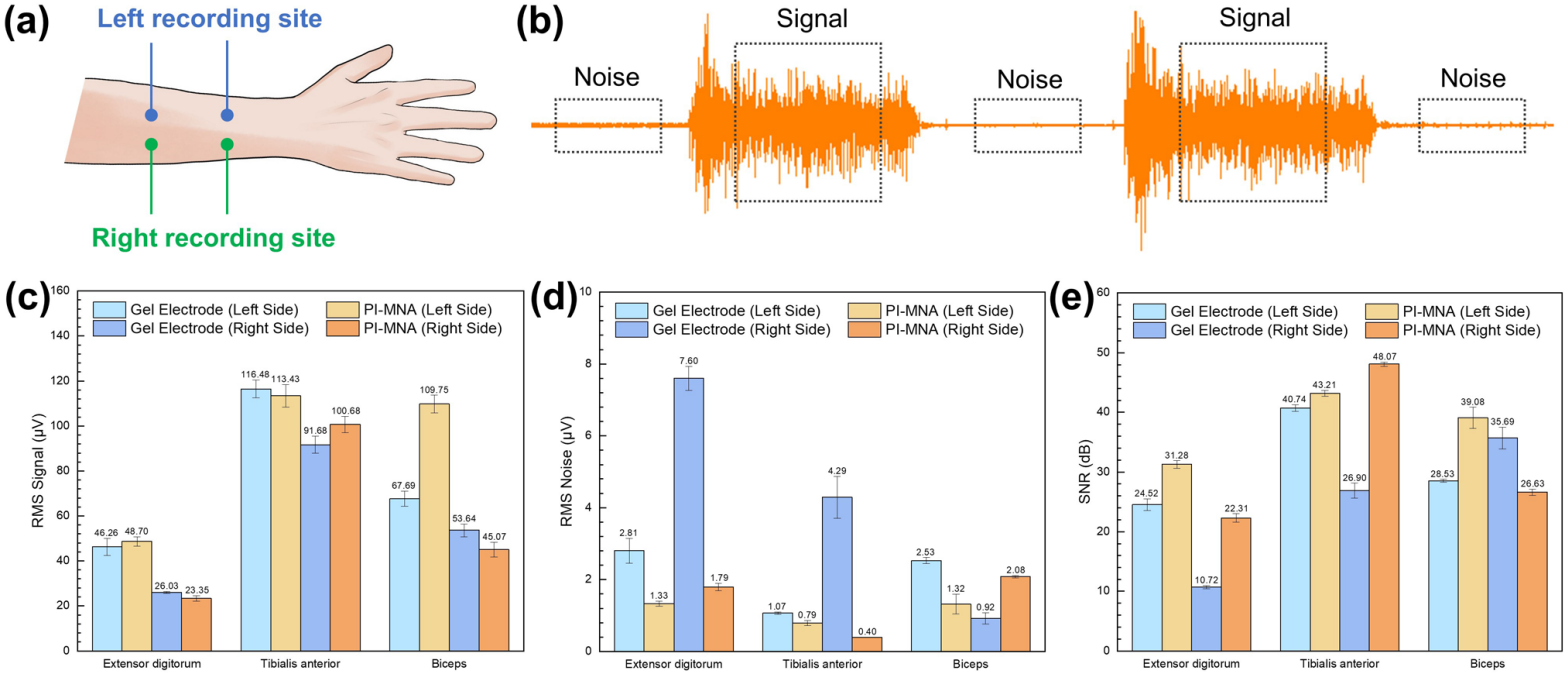
**a** Recording sites arrangement for SNR comparison between the PI-MNA and gel electrodes. **b** Schematic of the extraction for signal and noise segments for SNR analysis. **c** RMS signal, **d** RMS noise, and **e** SNR values calculated from the EMG signals recorded by two electrode types (*n* = 5, mean ± standard error)

First, the SNR performance of the PI-MNA and gel electrodes was compared using the EMG channels of the PSG instrument. As shown in Fig. 7a, two pairs of electrodes of different types were worn on the “left” and “right” sites of the same muscle to simultaneously record EMG signals. The signal and noise segments were selected (Fig. 7b), and the RMS and SNR were calculated. According to Fig. 7c–e, SNRs were analyzed from three muscles. The results show that the PI-MNA electrodes obtained higher SNR properties in most cases, proving the superiority of the PI-MNA electrodes in biopotential recording once again.

**Fig. 8:**
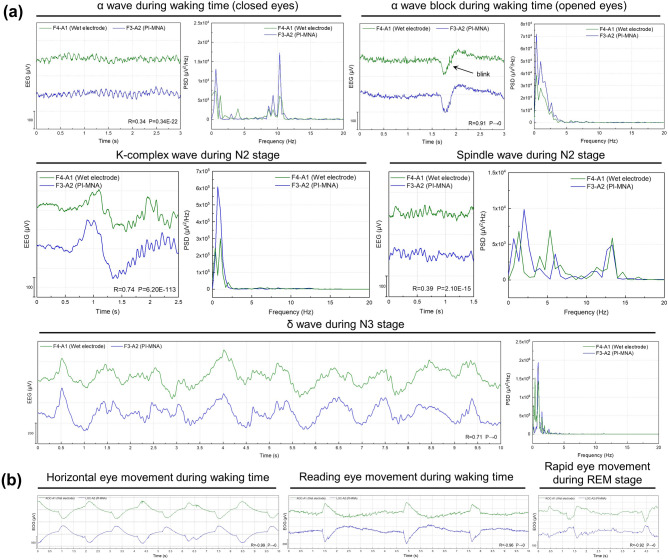
**a** Time and frequency domains of typical frontal EEG waveforms recorded by the PI-MNA and wet electrodes at symmetrical recording sites. (PSD: power spectral density. R: Pearson correlation coefficient. P: P value of Pearson correlation analysis. F3, F4, A1, A2: standard EEG recording sites defined by the international 10–20 system) **b** Typical EOG waveforms recorded by the PI-MNA and wet electrodes at symmetrical recording sites. (ROC: right outer canthus. LOC: left outer canthus)

For actual sleep monitoring, a volunteer wore the PI-MNA and wet electrodes at symmetrical recording sites overnight. The standard electrode locations of PSG are shown in Fig. S9. Figure 8a shows the typical waveforms of frontal EEG recorded in different states. The corresponding frequency analysis is also consistent with the characteristics of the waveforms. Figure 8b shows the typical waveforms of EOG. The correlation coefficients of the EEG and EOG from the two electrode types were calculated, showing a high correlation for each waveform. Furthermore, the volunteer wore only the PI-MNA electrodes for sleep monitoring again. Typical waveforms with high quality and high bilateral correlation were obtained (Fig. S10).

**Fig. 9:**
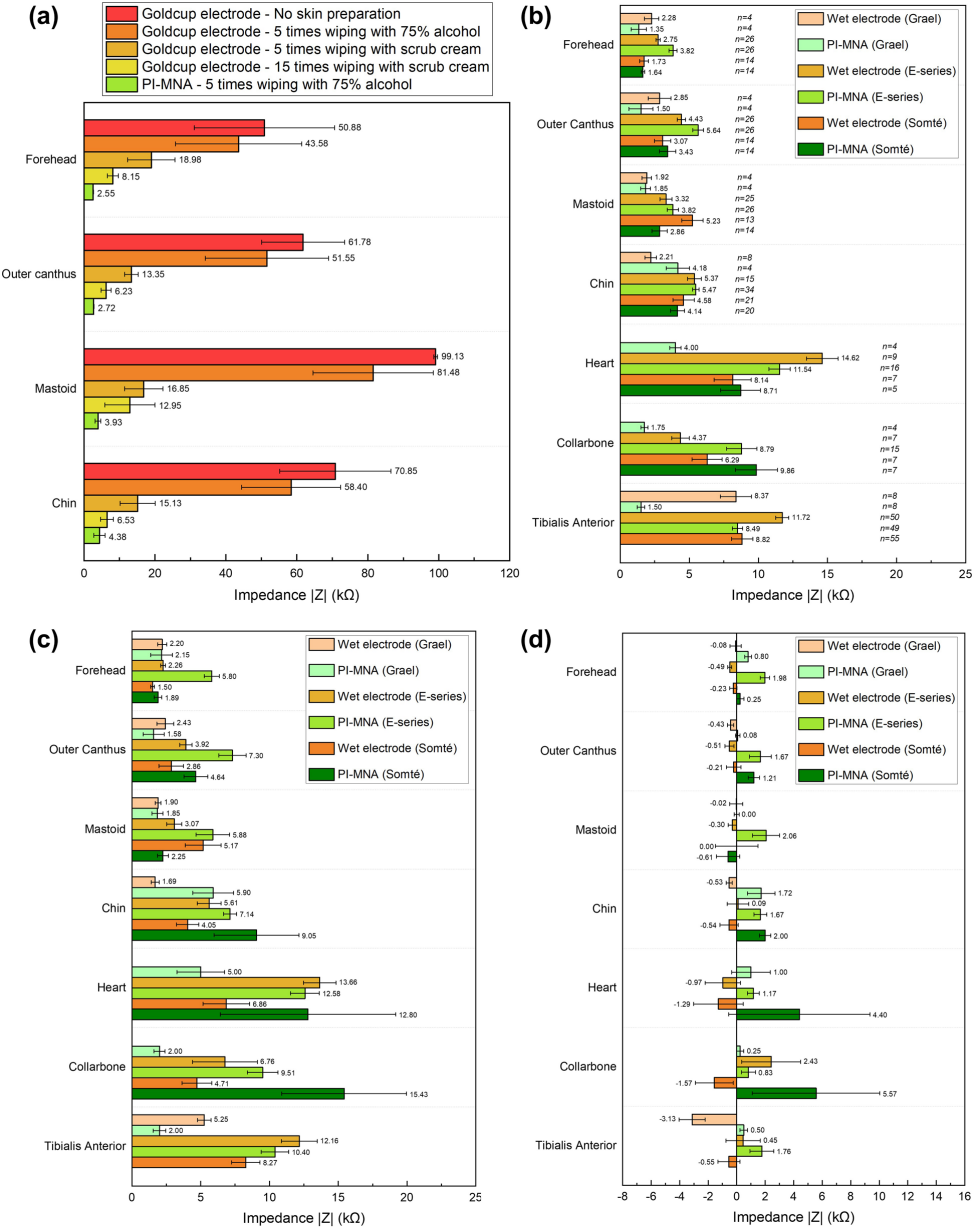
**a** EII property (1 kHz) of wet electrodes under different skin preparation conditions compared to that of the PI-MNA electrodes without skin preparation at four key locations of PSG (*n* = 4, mean ± standard error). Average EII (1 kHz) of electrodes **b** before and **c** after whole-night recording. **d** The changed values of EII (1 kHz) at the end of monitoring compared to those before monitoring. The number of electrodes included in the statistics of each case is provided in b (mean ± standard error)

Furthermore, a clinical study of the PI-MNA electrodes in PSG was carried out. To characterize the monitoring accuracy of the PI-MNA electrodes, the influence of EII on signal quality should be considered. The EIIs of wet electrodes under different skin preparation conditions were compared with those of the PI-MNA electrodes. As shown in Fig. 9a, skin preparation, especially by scrub cream, is critical to reducing the EII of wet electrodes. The purpose of wiping 75% alcohol before wearing the PI-MNA electrodes was to disinfect the skin surface.

In the clinical study, 12 healthy subjects and 16 subjects with OSAHS symptoms were enrolled. Figure 9b–d provides a comparison of EII before and after whole-night recording. The EII of wet electrodes generally remained stable due to the continuous infiltration of the conductive gel into skin. The PI-MNA electrodes were limited by the current method of simple fixation with medical tape; thus, the EII increased slightly because the subjects might have autonomous or involuntary movements during sleep. The EIIs of ECG and leg EMG sites with larger motion ranges exhibited more significant changes. Nevertheless, the PI-MNA electrodes maintained a low EII level at the end of the monitoring and thus did not result in any failure of recording. Note that some subjects’ movements during overnight sleep would inevitably result in severe loosening (> 50 kΩ) or failing (> 100 kΩ) of very few electrodes, whether wet or PI-MNA electrodes. These electrodes were not included in the EII analysis in Fig. 9b–d and were separately analyzed in Supplementary Fig. S11a. For EEG, EOG, and Chin EMG electrodes, which are key to sleep staging, the PI-MNA electrodes showed only one loosening at the Chin EMG site, while goldcup electrodes failed four times and loosened once, including one failing and one loosening that occurred at the mastoid, which could cause EEG and EOG signals to be unreadable. ECG and Leg EMG electrodes showed high levels of abnormality, mainly due to the further distance to the PSG instrument, the longer wires, and more movements. Compared to gel electrodes, the abnormal proportion of the PI-MNA electrodes is higher. The large adhesive area of the gel electrode did have an advantage in fastness, but the button connectors were sometimes were disconnected due to the movements, which increased the proportion of failing in abnormal gel electrodes.

**Fig. 10:**
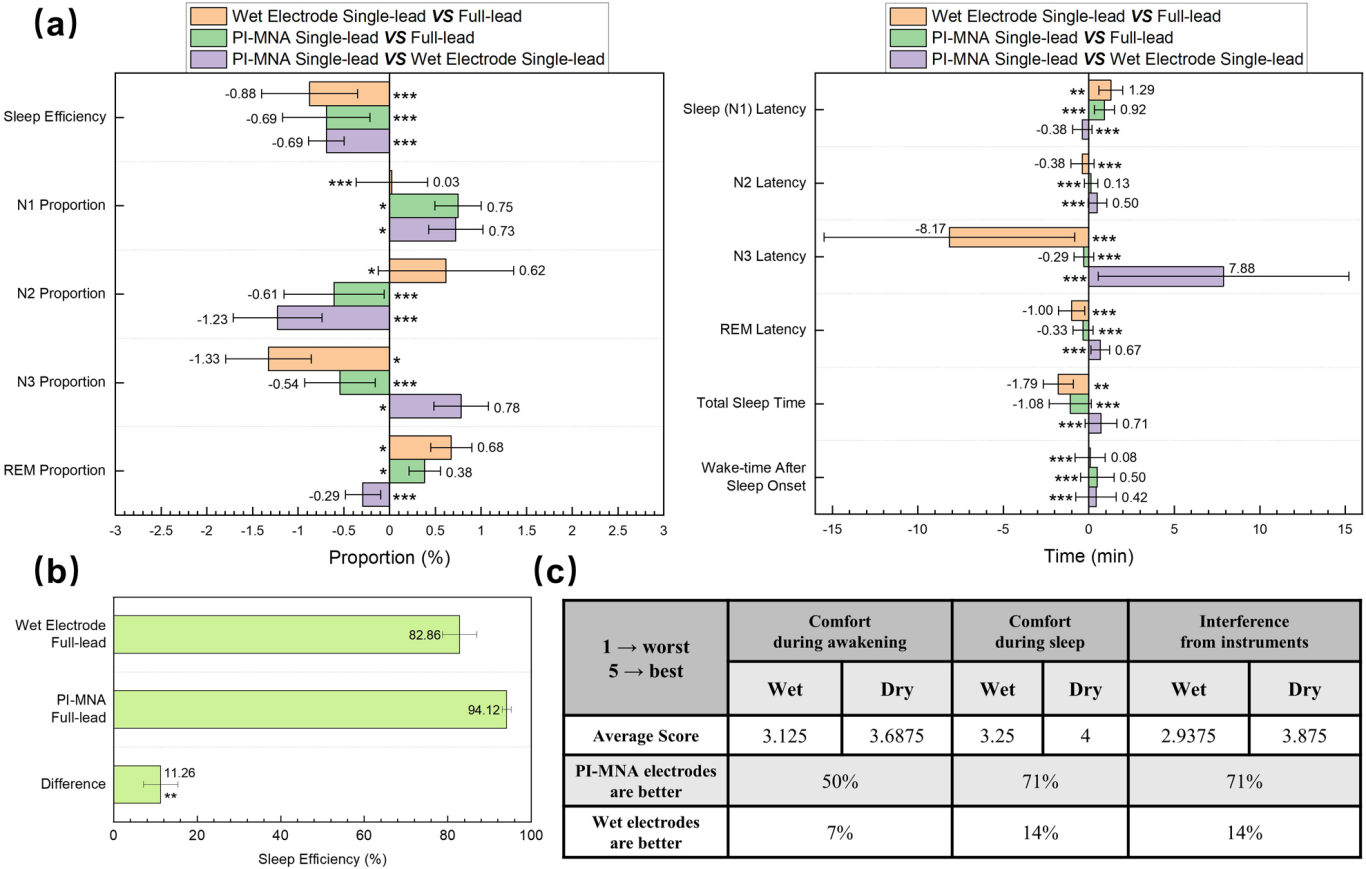
**a** Sleep indicators compared using data from all leads, data from single-side wet electrode leads, and data from single-side PI-MNA electrode leads (*n* = 12, mean ± standard error). “***”, “**”, and “*” indicate *p* > 0.1, *p* > 0.05, and *p* > 0.01 in the paired-samples t test. **b** Sleep efficiency analyzed from PSG data recorded by two electrode types during two nights of sleep monitoring for OSAHS subjects (*n* = 16, mean ± standard error). “**” indicates *p* > 0.05 in the paired-samples t test. **c** Subjective evaluation of the sleep monitoring comfort with two electrode types

The accuracy of PSG is reflected in the indicators analyzed from the recorded data. For the healthy subject study, 11 sleep indicators were compared. As the statistical analysis shown in Fig. 10a, sleep indicators obtained by the three configurations were very similar, with the difference in sleep efficiency (SE) and sleep stage proportion within 2%, as well as the difference in latency, total sleep time (TST), and wake-time after sleep onset (WASO) was basically within 2 min. Moreover, t tests were used to analyze the sleep indicators of each pair of configurations, proving no significant difference. Note that one of the N3 latency data points from wet electrode leads showed a large deviation, and the difference was significantly reduced when the data point was removed (Fig. S11b). The statistical conclusion indicates that accurate sleep interpretation can be achieved when EEG data are recorded only from a single-side frontal lead by the PI-MNA electrodes. Furthermore, PSG data from OSAHS subjects were used to characterize the difference in comfort between two nights of monitoring using wet and PI-MNA electrodes. Figure 10b shows the SE analyzed from PSG data from two electrode types. When the PI-MNA electrodes were employed, the subjects’ average SE increased by 11.26%. Moreover, in Fig. 10c, the subjective evaluation from the subjects’ questionnaires of three items indicated that the PI-MNA electrodes were more comfortable. In the line of average score, “1” means the worst and “5” means the best comfort fell. The proportions in the last two lines show whether the subjects feel better wearing wet or PI-MNA electrodes for sleep monitoring. The results showed that 50%, 71%, and 71% of the subjects considered the PI-MNA electrodes were better in the three items, compared to only 7%, 14%, and 14% of the subjects who considered wet electrodes were better. The remaining subjects could not easily judge.

As a conclusion of the clinical study, the PI-MNA electrodes have accordant sleep monitoring ability as the standard wet electrodes and have superior convenience when wearing, as well as giving users better comfort without the employment of conductive gel. Therefore, the PI-MNA electrodes have sufficient substitution capability for wet electrodes during overnight PSG application.

## Conclusion and Discussion

The PI-MNA electrodes proposed in this work are very suitable for wearable biopotential recording. As a novel flexible dry electrode, the low EII, high mechanical strength, good biosafety, small footprint, comfortable wearing, and controllable cost of the PI-MNA represent significant advantages over standard wet electrodes and existing research results.

Owing to the microneedle structure and surface modification, the normalized EII of the PI-MNA electrodes reaches 0.98 kΩ cm^2^ at 1 kHz and 1.50 kΩ cm^2^ at 10 Hz, a record low value compared to previous reports, to the best of our knowledge, and only approximately 1/250 of the standard wet electrodes. The minimized footprint with low impedance enables high spatial resolution and high SNR. The evaluation of the contact area in this work is based on the footprint that covered by the electrode on the skin surface, and does not calculate the surface area of microneedles. In fact, the three-dimensional microneedle structure offers more low-impedance contact area, which is one of the significant advantages of the MNA electrode. Certainly, a higher microneedle, denser array, and larger surface area can result in better electrode–skin contact under the same footprint. However, the mechanical strength and the risk of skin injury caused by excessively high microneedles should also be considered.

The material and structure of the PI-MNA electrodes simultaneously achieve flexibility of the substrate to fit the curved body surface and robustness of microneedles to penetrate the skin without fracture. No fracture occurred after 100 penetrations to human skin. To date, thousands of PI-MNA electrodes have been applied to the human body without causing inflammation, allergies or other adverse reactions. Microneedles caused only a slight prickling sensation when attached to skin, and the penetration marks usually spontaneously faded within hours. The simplified process allows mass production with a cost down to $0.35 for each PI-MNA electrode.

Combined with the tag-style wearable wireless recording system, the PI-MNA electrodes showed good signal quality. We quantified the advantages of the PI-MNA electrodes in terms of motion artifacts and SNR. The sufficiently small footprint and nongel structure allow the PI-MNA electrodes to be directly attached to wearable tag-style systems containing biopotential acquisition and wireless transmission circuits (Fig. S1c), achieving nonfeeling wearing for patients without disturbing sleep or other daily activities. Due to the nonrequest of skin preparation, patients can wear and remove the electrodes and tags by themselves without the help of a professional operator. The preparation time can be reduced from over 30 min (for commercial instruments and wet electrodes) to just several minutes, which significantly improves the convenience of electrophysiological recording. The lightweight tag-style systems also avoid the risk of the electrode falling off.

The performance of the PI-MNA electrodes on an overnight time scale application was demonstrated in the clinical study of PSG by a standard instrument. Tens of healthy and sleep-disordered subjects participated in the study and provided 44 nights of recording (over 8 h per night). Through the statistical analysis of EII, sleep indicators, and user comfort, sufficient substitution of the PI-MNA electrodes for clinical standard wet electrodes has been proven.

Nevertheless, for PSG or other electrophysiological recording instruments, the large instrument and mass wires bring more uncomfortable sensation than the electrodes, which affects the accuracy of monitoring. Therefore, wearable and wireless tag-style systems with PI-MNA electrodes are a much better solution to solve this problem and achieve nonfeeling wearing. Ongoing studies are aimed at better integrated design of the PI-MNA electrodes and wearable circuits, as well as toward higher electrode densities, since multiple small-sized PI-MNA electrodes can be placed closely without fear of shorting due to the needlessness of conductive gel. In conclusion, the successful development and verification of the PI-MNA electrode effectively cleared the obstacles in comfortable and efficient wearability, high temporal and spatial resolution, and long-term application.

## Supplementary Information

Below is the link to the electronic supplementary material.

Supplementary file1 (PDF 1436 KB)
