# Supplementary material for: High-Performance Flexible Microneedle Array as a Low-Impedance Surface Biopotential Dry Electrode for Wearable Electrophysiological Recording and Polysomnography
Source: Nanomicro Lett. 2022 Jun 14;14:132. doi: 10.1007/s40820-022-00870-0 (PMC9198145; doi:10.1007/s40820-022-00870-0)
Supplement: Supplementary file 1 — Supplementary file1 (PDF 1436 KB) [file 40820_2022_870_MOESM1_ESM.pdf]

Supporting Information for

# High-Performance Flexible Microneedle Array as a Low-Impedance Surface Biopotential Dry Electrode for Wearable Electrophysiological Recording and Polysomnography

Junshi Li[1], Yundong Ma[2], Dong Huang[1,3,4], Zhongyan Wang[1], Zhitong Zhang[1], Yingjie Ren[1], Mengyue Hong[2], Yufeng Chen[1], Tingyu Li[1], Xiaoyi Shi[1], Lu Cao[1, 5], Jiayan Zhang[1], Bingli Jiao[3], Junhua Liu[1], Hongqiang Sun[2, *], Zhihong Li[1, *]

[1]National Key Laboratory of Science and Technology on Micro/Nano Fabrication, School of Integrated Circuits, Peking University, Beijing 100871, People's Republic of China

[2]Peking University Sixth Hospital, Peking University Institute of Mental Health, NHC Key Laboratory of Mental Health (Peking University), National Clinical Research Center for Mental Disorders (Peking University Sixth Hospital), Beijing 100191, People's Republic of China

[3]School of Electronics, Peking University, Beijing 100871, People's Republic of China

[4]Hypnometry Microsystem, Beijing 100871, People's Republic of China

[5]College of Engineering, Peking University, Beijing 100871, People's Republic of China

*Corrresponding authors. E-mail: zhhli@pku.edu.cn (Zhihong Li), sunhq@bjmu.edu.cn (Hongqiang Sun)

## Supplementary File S1 - PSG Comfort Questionnaire

**Rate the comfort of the device while AWAKE:**

1. Extremely uncomfortable.
2. Moderately uncomfortable.
3. Mildly uncomfortable.
4. Minimally uncomfortable.
5. Comfortable.

**Rate the comfort of the device while SLEEPING:**

1. Extremely uncomfortable.
2. Moderately uncomfortable.
3. Mildly uncomfortable.
4. Minimally uncomfortable.
5. Comfortable.

**Rate how much the device disturbed your sleep:**

1. Extremely disturbed compared to my usual sleep.
2. Moderately disturbed compared to my usual sleep.

3. Mildly disturbed compared to my usual sleep.
4. Minimally disturbed compared to my usual sleep.
5. My sleep was typical of my usual sleep (no interference)

## S2 Supplementary Figures

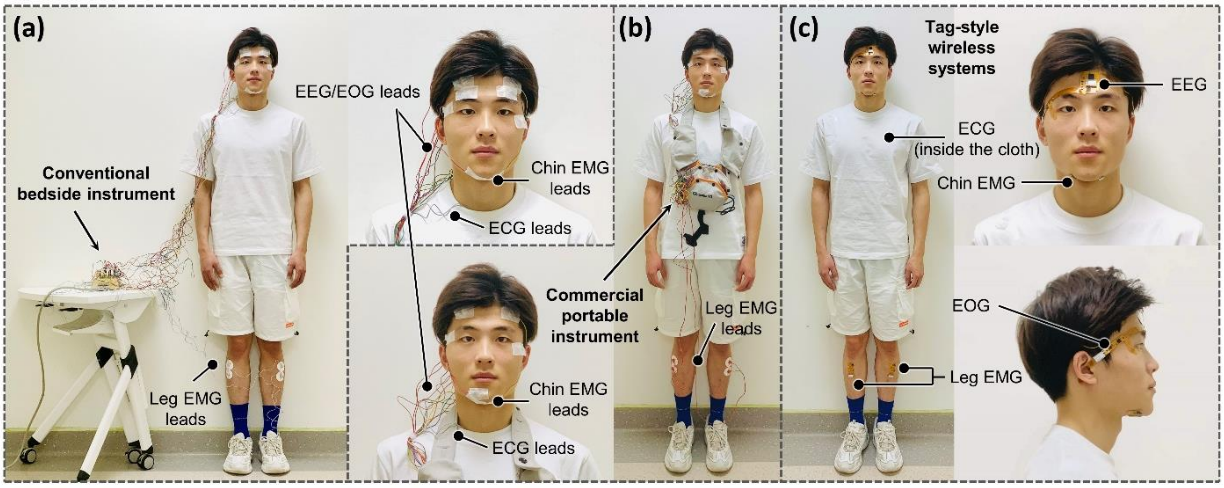


**Fig. S1** Photographs of a volunteer wearing standard PSG instruments with wet electrodes and wireless recording systems with PI-MNA electrodes. **a** Conventional bedside PSG instrument, Compumedics E-series (electrophysiological leads only). **b** Commercial portable PSG instrument, Compumedics Somté (electrophysiological leads only). **c** Tag-style wireless recording systems

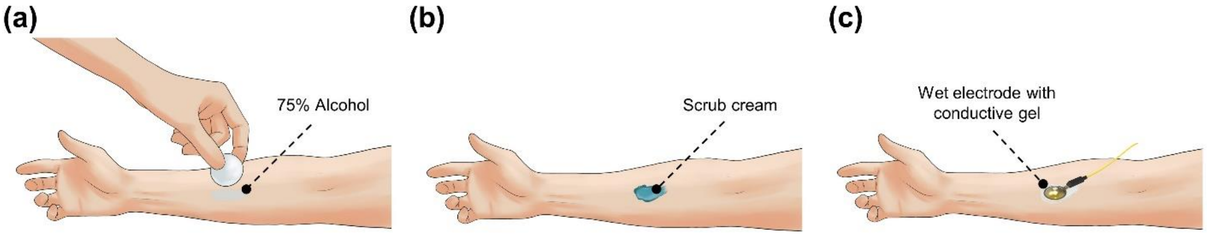


**Fig. S2** Skin preparation procedure before applying wet electrode on skin. **a** The skin around the recording site is wiped with 75% alcohol to remove grease and disinfect. **b** The skin around the recording site is wiped with scrub cream to thin the stratum corneum. **c** Fill the goldcup wet electrode with conductive gel and press firmly on the treated skin

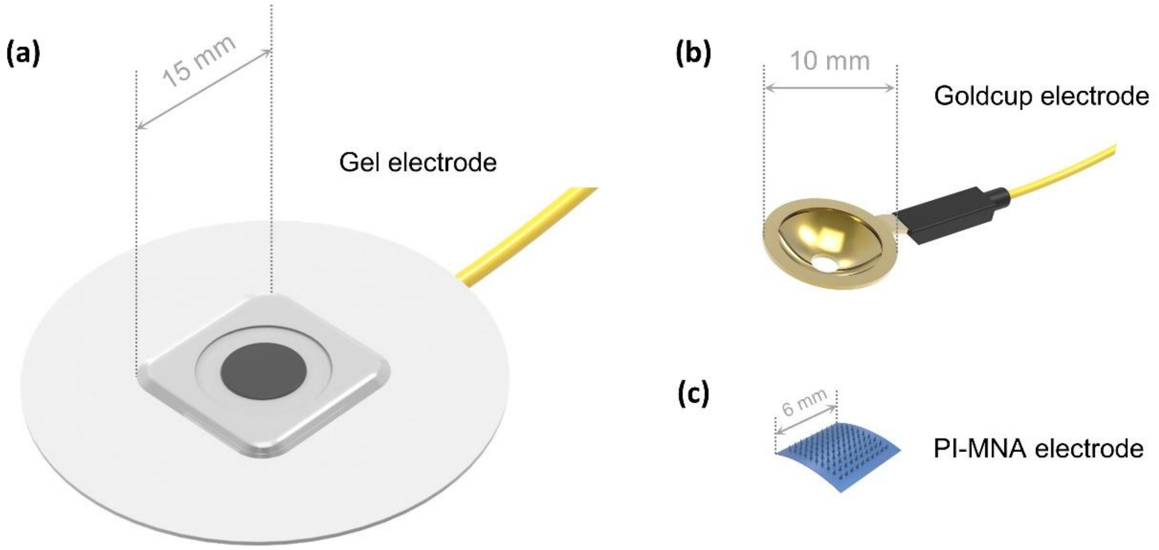


**Fig. S3** Schematic of clinical standard wet electrodes and the PI-MNA electrode. **a** Gel electrode that has conductive gel prefixed to an Ag/AgCl plate, which is often led out to the external device through a button-type connector on the back side. **b** Goldcup electrode that has a cup shape and is plated with gold. The "cup" needs to be filled with conductive gel to form a good electrode-skin contact when used. **c** The PI-MNA electrode (PEDOT:PSS modified) proposed in this work

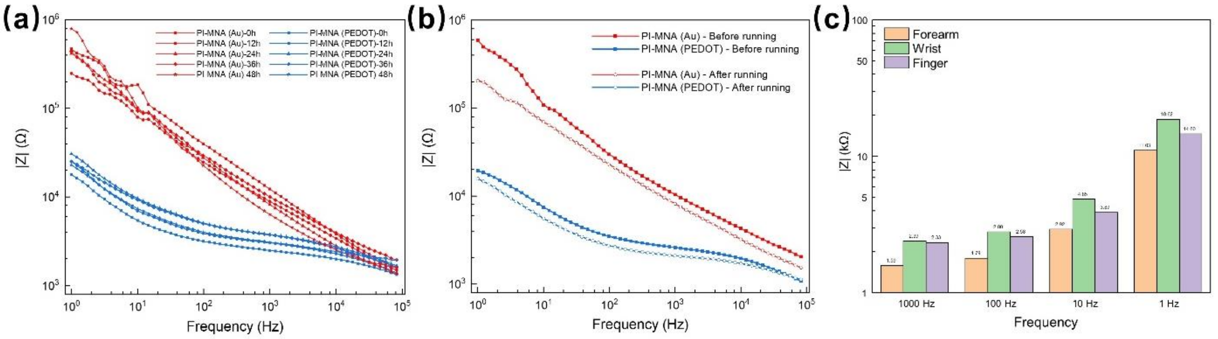


**Fig. S4** EII stability of the PI-MNA electrode. **a** EII characterization every 12 h during 48 h of continuous electrode wearing. **b** EII characterization in a sweating state when a volunteer was required to run outdoors for 30 minutes while wearing the electrodes. **c** EII measured on a significantly curved skin surface

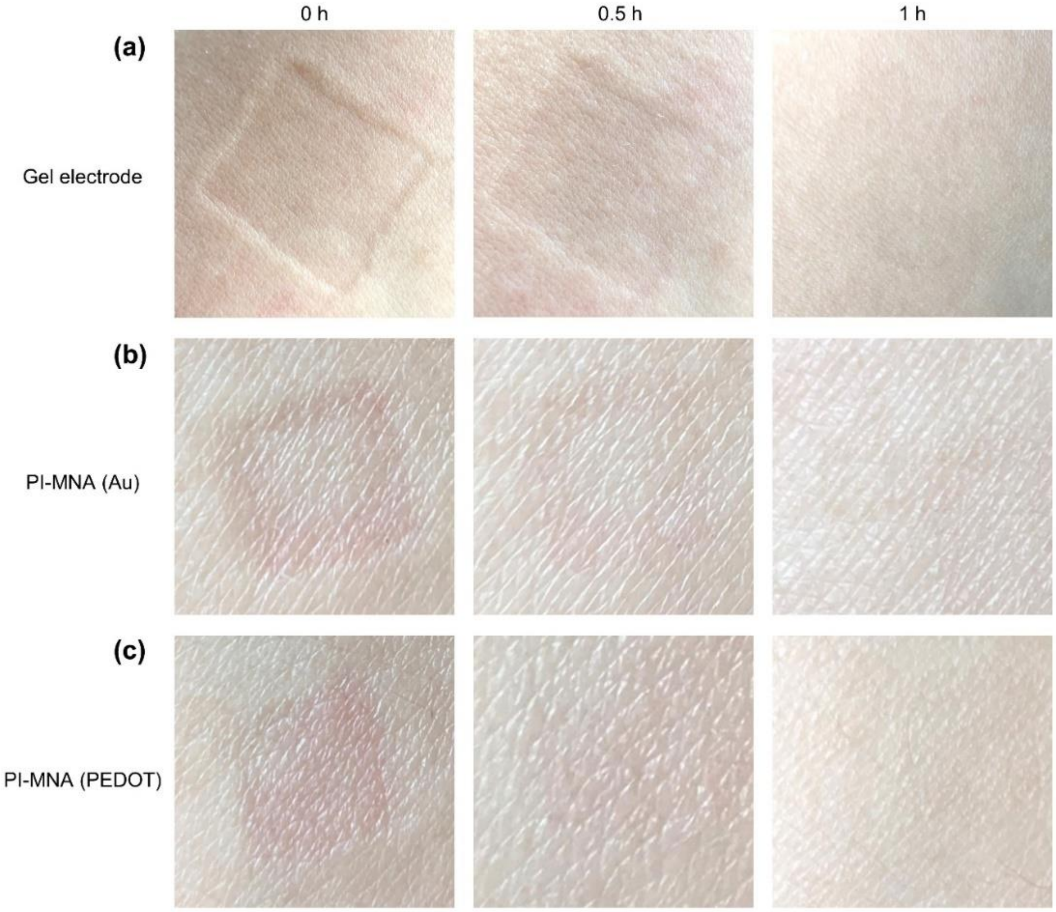


**Fig. S5** Skin reaction characterization to verify the biocompatibility of the PI-MNA electrode. Photographs of the electrode wearing positions immediately, 0.5 h, and 1 h after removing the **a** gel electrode, **b** gold-surface PI-MNA electrode, and **c** PEDOT:PSS-surface PI-MNA electrode that were continuously worn for 2 hours

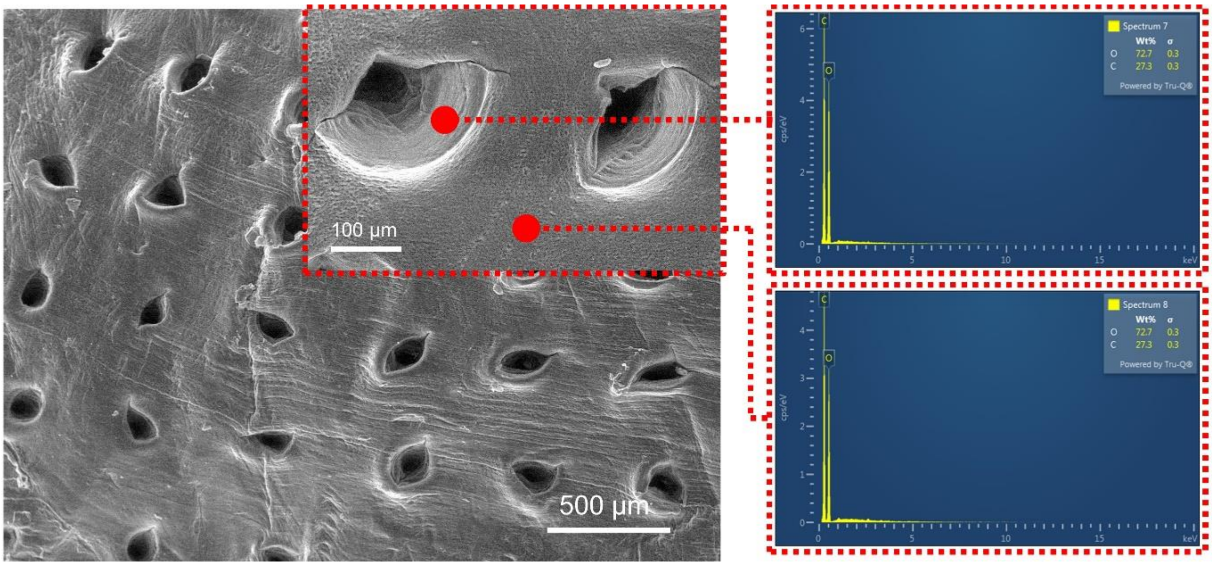


**Fig. S6** SEM micrograph and EDS analysis of the agarose gel substrate after the inserting/retracting PEDOT:PSS modified PI-MNA electrode

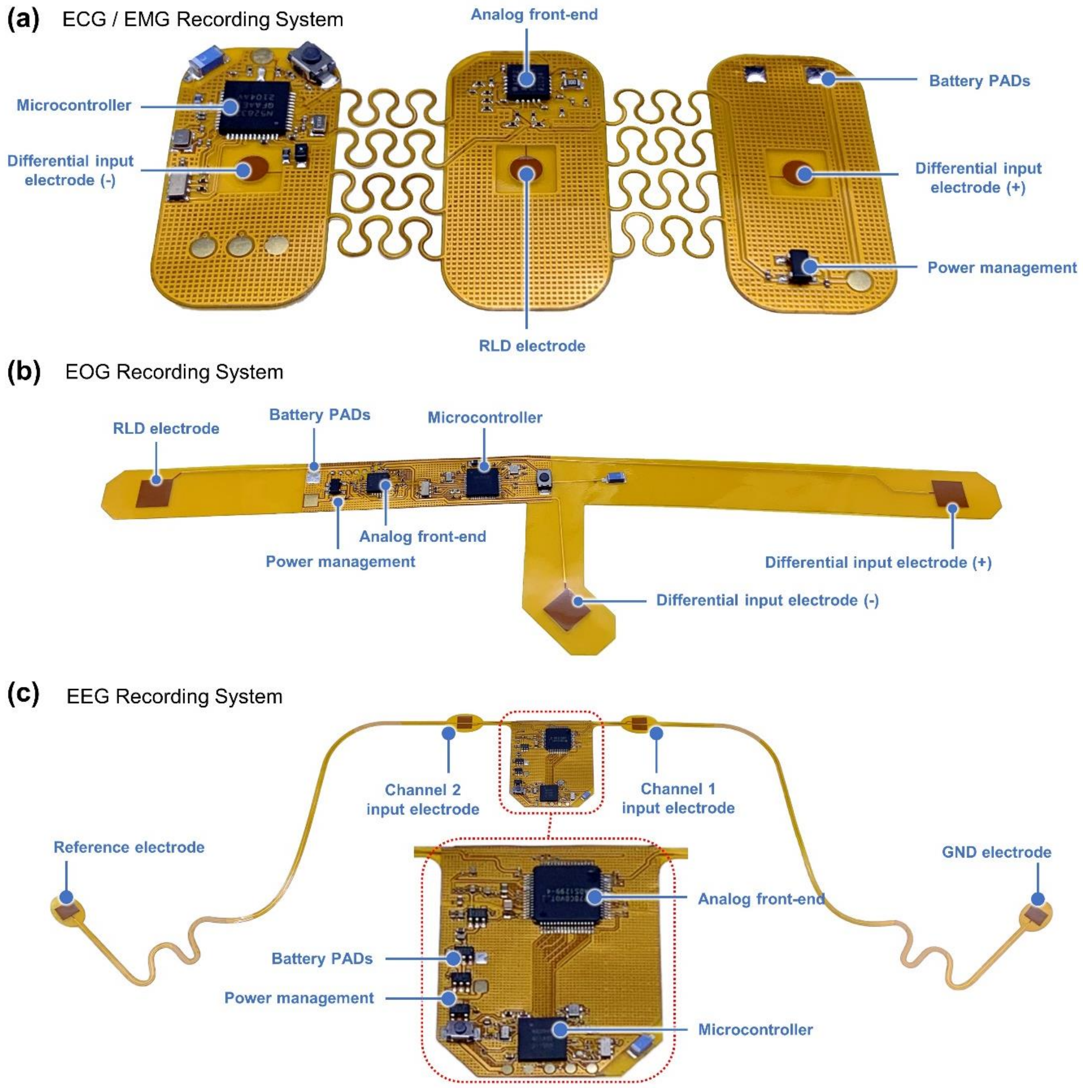


**Fig. S7** Wearable wireless recording systems based on flexible printed circuit (FPC) board. **a** ECG and EMG recording system with serpentine stretchable lines. **b** EOG recording system with wearable design. **c** Two-channel frontal EEG recording system with wearable design

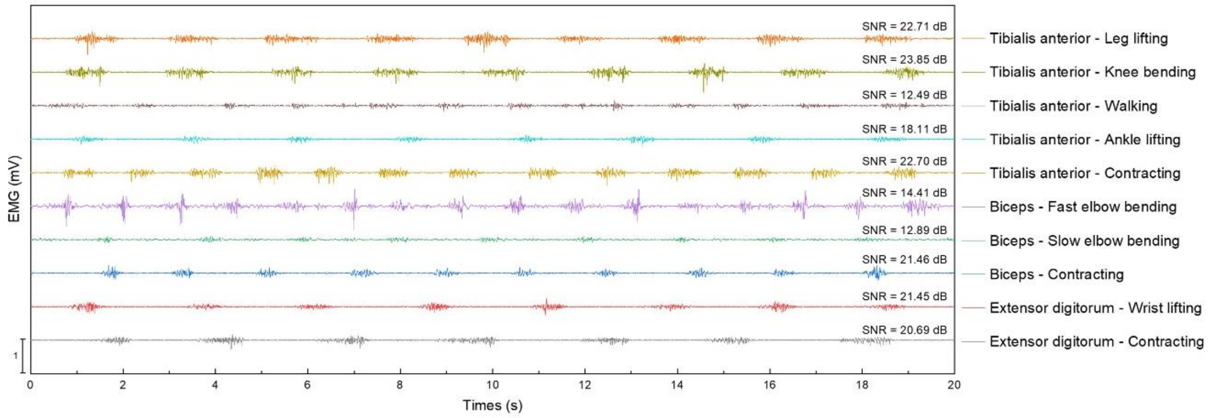


**Fig. S8** EMG recorded during 10 different muscle or joint movements via wearable wireless recording system and the PI-MNA electrodes. High-pass (20 Hz) and harmonic comb (50 Hz) digital filtering are applied to eliminate low-frequency artifacts and power frequency interference, respectively. The SNR values are the same as those in Fig. 5g

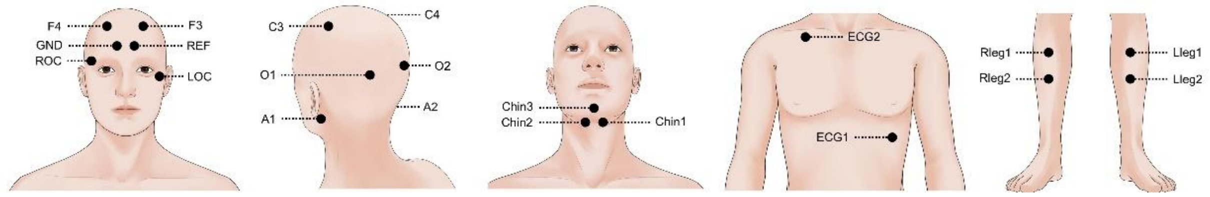


**Fig. S9** Standard electrode locations of PSG for electrophysiological signal recording. F3, F4, C3, C4, O1, O2, A1, A2: EEG recording and reference sites defined by the international 10–20 system. ROC, LOC: EOG recording sites near the right and left outer canthus. Chin1, Chin2, Chin3: Chin EMG recording and reference sites (the instrument type Compumedics E-series does not have the Chin3 site, and single-lead Chin EMG is recorded from Chin1-Chin2). ECG1, ECG2: ECG recording site near the apex cordis and reference site near the collarbone. Rleg1, Rleg2, Lleg1, Lleg2: Tibialis Anterior EMG recording and reference sites at right and left leg

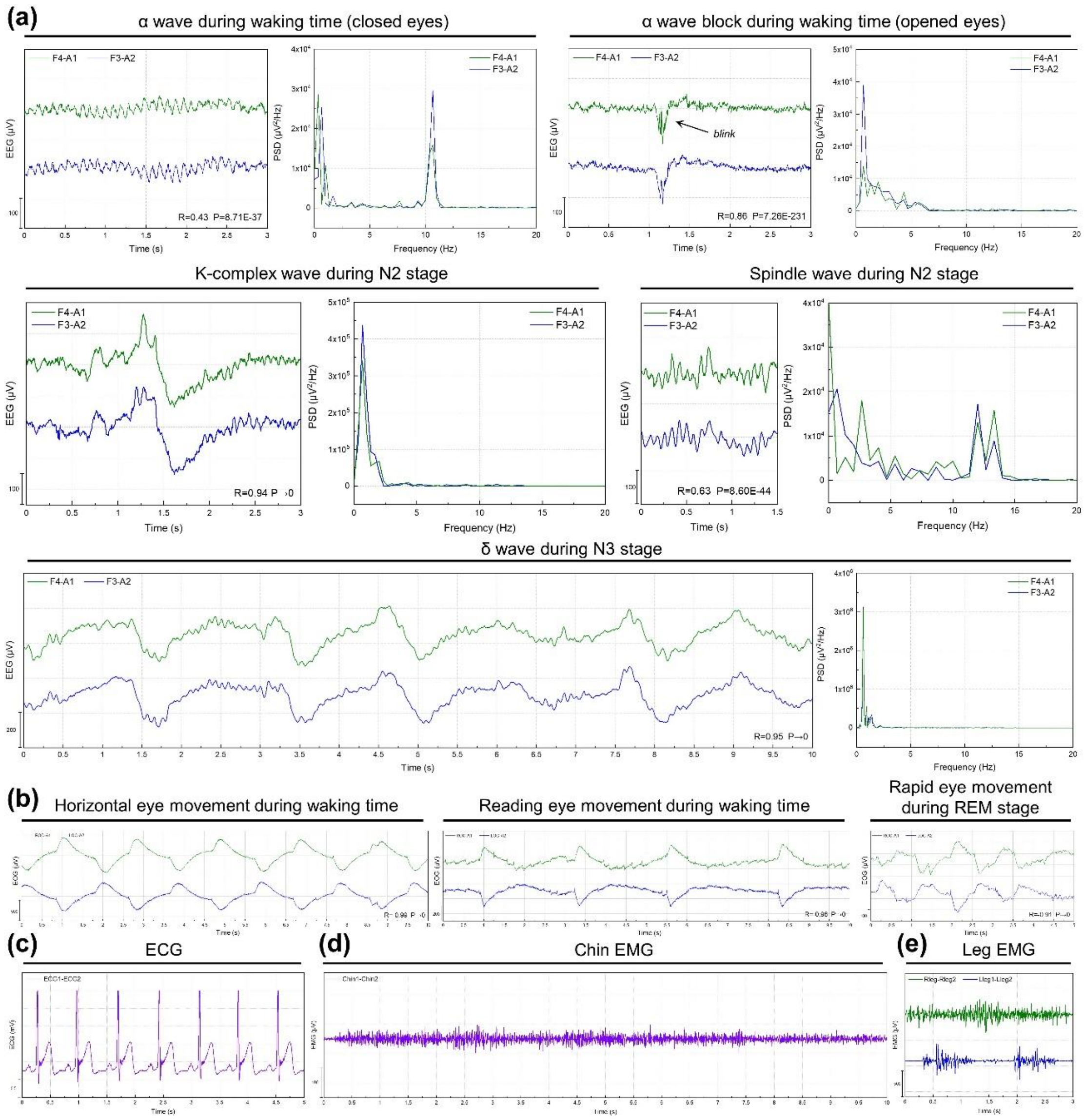


**Fig. S10** Typical waveforms recorded by PI-MNA electrodes at symmetrical recording sites. **a** Frontal EEG signals. **b** EOG signals. **c** ECG signals. **d** Chin EMG signal. **e** Leg EMG signals. (PSD: power spectral density. R: Pearson correlation coefficient. P: Pvalue of Pearson correlation analysis)

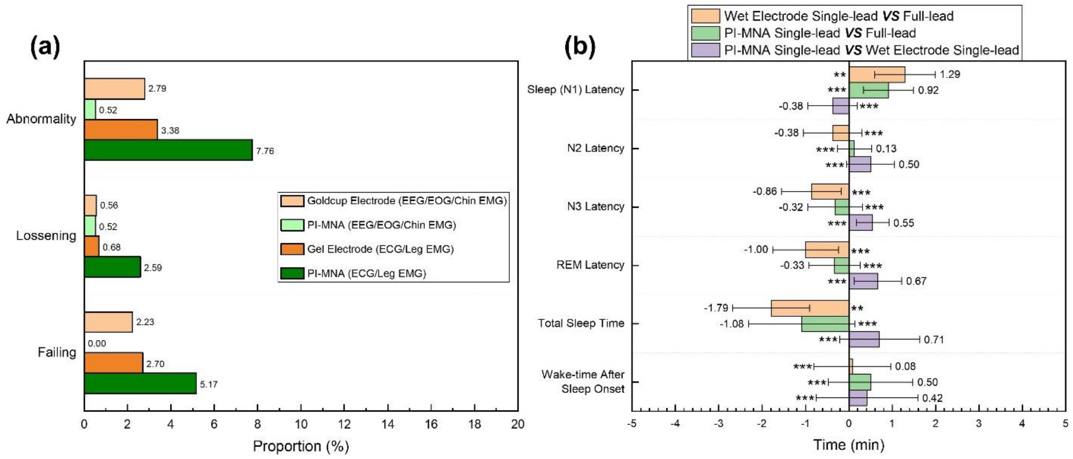


**Fig. S11** Supplementary analysis in the clinical PSG study for PI-MNA electrodes. **a** Proportion of abnormal electrodes in all 44 nights of PSG. "Loosening" and "Failing" indicate the EII at the end of the monitoring over 50 kΩ and 100 kΩ at 1 kHz, respectively. "Abnormality" is the sum of "Loosening" and "Failing". **b** Diagram with the same meaning as Fig. 10a (right diagram) that removed a data point with a large deviation of N3 latency
